# Interactions between Rainbow Trout Eyed Eggs and *Flavobacterium* spp. Using a Bath Challenge Model: Preliminary Evaluation of Bacteriophages as Pathogen Control Agents

**DOI:** 10.3390/microorganisms9050971

**Published:** 2021-04-30

**Authors:** Valentina L. Donati, Inger Dalsgaard, Anniina Runtuvuori-Salmela, Heidi Kunttu, Johanna Jørgensen, Daniel Castillo, Lotta-Riina Sundberg, Mathias Middelboe, Lone Madsen

**Affiliations:** 1 Unit for Fish and Shellfish Diseases, National Institute of Aquatic Resources, Technical University of Denmark, 2800 Kongens Lyngby, Denmark; inda@aqua.dtu.dk (I.D.); loma@aqua.dtu.dk (L.M.); 2 Department of Biological and Environmental Science and Nanoscience Center, University of Jyväskylä, P.O. Box 35, FI-40014 Jyväskylä, Finland; anniina.runtuvuori@jyu.fi (A.R.-S.); heidi.kunttu@jyu.fi (H.K.); lotta-riina.sundberg@jyu.fi (L.-R.S.); 3 Marine Biological Section, Department of Biology, University of Copenhagen, 3000 Helsingør, Denmark; johanna.jorgensen@bio.ku.dk (J.J.); daniel.castillo@bio.ku.dk (D.C.); mmiddelboe@bio.ku.dk (M.M.)

**Keywords:** *Flavobacterium psychrophilum*, *Flavobacterium columnare*, rainbow trout, eyed eggs, phage-mediated control, bacteriophages

## Abstract

The microbial community surrounding fish eyed eggs can harbor pathogenic bacteria. In this study we focused on rainbow trout (*Oncorhynchus mykiss*) eyed eggs and the potential of bacteriophages against the pathogenic bacteria *Flavobacterium psychrophilum* and *F. columnare*. An infection bath method was first established, and the effects of singular phages on fish eggs was assessed (survival of eyed eggs, interaction of phages with eyed eggs). Subsequently, bacteria-challenged eyed eggs were exposed to phages to evaluate their effects in controlling the bacterial population. Culture-based methods were used to enumerate the number of bacteria and/or phages associated with eyed eggs and in the surrounding environment. The results of the study showed that, with our infection model, it was possible to re-isolate *F. psychrophilum* associated with eyed eggs after the infection procedure, without affecting the survival of the eggs in the short term. However, this was not possible for *F. columnare*, as this bacterium grows at higher temperatures than the ones recommended for incubation of rainbow trout eyed eggs. Bacteriophages do not appear to negatively affect the survival of rainbow trout eyed eggs and they do not seem to strongly adhere to the surface of eyed eggs either. Finally, the results demonstrated a strong potential for short term (24 h) phage control of *F. psychrophilum*. However, further studies are needed to explore if phage control can be maintained for a longer period and to further elucidate the mechanisms of interactions between Flavobacteria and their phages in association with fish eggs.

## 1. Introduction

The physical barrier of the thin chorion (*zona pellucida*) and the thicker inner membrane (*zona radiata*) of teleost eggs varies in structure and thickness among species [1], and represents the first line of defense against bacterial and viral infections. The wide range of the bacteria that surrounds the eggs will contribute to the early establishment of the fish microbiome [2,3]. Within these microbial communities, pathogenic bacteria such as *Cytophaga* spp., *Flavobacterium* spp., *Vibrio* spp., *Pseudomonas* spp., and *Aeromonas* spp. also exist, and may represent threats for the development and survival of the fish [3,4,5,6]. In aquaculture facilities, egg disinfection protocols are used to decrease the risk of mortality and pathogen transmission [7].

The transmission of the freshwater pathogen *Flavobacterium psychrophilum* [8,9], an etiological agent of rainbow trout fry syndrome (RTFS) and bacterial coldwater disease (BCWD), among fish populations is not fully understood. Both the vertical and the horizontal routes have been suggested to play a role [10,11,12], and *F. psychrophilum* has been isolated from milt, ovarian fluids, and in close connection with eggs [12,13,14], as well as from the surrounding environment of diseased fish [12,15]. Similarly to *F. psychrophilum*, the freshwater pathogen *F. columnare*, which causes mortality in wild and culture freshwater fish, characterize the microbial communities of fish, eggs, and the rearing waters (reviewed by [16]). Persistent colonization of eggs by Flavobacteria thus likely increase the probability of bacterial transmission to fish in all production stages, which can lead to important economic losses and the increased use of antibiotics [16]. Both *F. psychrophilum* and *F. columnare* cause high mortalities in rainbow trout fry populations (up to 80–90%), depending on the size of the fish [17,18,19]. Good husbandry management and egg disinfection have been highlighted as methods to reduce the development of infections among fish in hatcheries [12].

The utilization of virulent bacteriophages (also called phages) [20] to reduce mortality and prevent the spread of bacterial populations among fish and crustaceans at different stages has gained increased attention (reviewed by [21,22]). Phage therapy is considered a potential alternative to antibiotics, aiming to reduce the issues related to the use of antibiotics, and as a preventive measure against the spread of bacterial infections (reviewed by [23]).

Previous studies on phage control of *Flavobacterial* pathogens in rainbow trout have focused on fry and juvenile stages [24,25,26,27,28]. Here, we report for the first time the use of bacteriophages for reducing these pathogens in connection with rainbow trout eyed eggs. In this work, we explored the potential of using virulent bacteriophages targeting *F. psychrophilum* and *F. columnare* as bacterial control agents in rainbow trout eyed eggs. At first, we established a bacterial challenge bath method (Section A), and secondly, we evaluated the effects of phage addition on eyed eggs (Section B). Subsequently, we exposed rainbow trout eyed eggs to phages to assess their efficiency in eliminating the target bacterium (Section C).

## 2. Materials and Methods

### 2.1. Bacteria

*Flavobacterium psychrophilum* 950106-1/1 and 160401-1/5N, Danish strains isolated from rainbow trout, were selected for the experiments. *F. psychrophilum* 950106-1/1 is a well-characterized strain isolated in 1995 (serotype Fd, virulent) [10,29,30,31], while *F. psychrophilum* 160401-1/5N was isolated in 2016 and recently characterized (serotype Th, virulent) [31]. An additional strain, *F. psychrophilum* FPS-S6 (serotype Th, virulent, isolated in 2017 in Sweden), was used for the production of high titer phage FPSV-D22 solutions since it was the most efficient host for phage proliferation [27,31]. The strains were stored at −80 °C in tryptone yeast extract salts (TYES) medium [32] and glycerol (15–20%). For phage analysis, *F. psychrophilum* 950106-1/1 (and *F. psychrophilum* 160401-1/5N for Exp. I section C) was inoculated in TYES broth (5 mL, referred as TYES-B) from a −80 °C stock, incubated for 48–72 h (15 °C; 100 rpm) and then streaked onto TYES agar (TYES-B with 1.1% agar, referred as TYES-A). Single colonies were then picked (3–4 days of incubation) and inoculated in TYES-B for 48 h [27]. For bath challenge experiments, the selected bacteria were prepared according to [30]. Specifically, 0.5 mL of a 72 h bacterial culture (5 mL) was transferred into 100 mL TYES-B and incubated at 15 °C. After 48 h of incubation, appropriate dilutions depending on the selected dose of infection were performed prior to the bath. CFU were counted before and after the infection procedure in duplicates.

Two virulent isolates were used in the studies with *F. columnare*: B480 and B185. Originally, both strains were isolated from fish farms during columnaris disease outbreaks in Finland. Strain B480 was isolated from rainbow trout in 2012, and belongs to the genetic group E [33]. B185 was isolated from rearing tank water in 2008 [34]. Bacterial cultures were stored at −80 °C with 10% glycerol and 10% fetal calf serum. For the experiments, bacteria were revived from −80 °C by inoculation into 5 mL of Shieh medium [35] and cultured overnight at 25 °C under constant agitation (120 RPM). Bacteria were enriched by subculturing (1:10) and incubating for 24 h. Bacterial cell density was measured as an optical density (OD, 595 nm; Multiscan FC Thermo Scientific, Ratastie, Finland) and colony forming units per ml (CFU mL^−1^) estimated based on our previously determined OD-CFU relationship (unpublished).

### 2.2. Bacteriophages

*F. psychrophilum*-targeting lytic bacteriophages FpV4 (isolated in 2005 in Denmark from water with feces samples, Podoviridae family) [36,37] and FPSV-D22 (isolated in 2017 in Denmark from fish tissue samples, Siphoviridae family) [27,31] were selected for the studies. Solutions of FpV4 and FPSV-D22 were purified (0.2 μm pore size sterile filter) and stored in SM buffer (8 mM MgSO_4_, 50 mM Tris-Cl [pH 7.5], 99 mM NaCl, 0.01% gelatin) and glycerol (15%) at −80 °C [31,36]. For the experiments in section B, phage high titer solutions were prepared from crude lysates following the infection of the strain 950105-1/1 (for FpV4 propagation) and of the strain FPS-S6 (for FPSV-D22 propagation) in TYES-B (MOI = 1). After incubation for 48–72 h, the lysed cultures were then centrifuged (5000× *g*, 10 min, 4 °C) and filtered with a 0.2 μm pore size sterile filter (Sterivex, Millipore; Merck KGaA, Darmstadt, Germany). For the experiments in section B and C, FpV4 and FPSV-D22 crude lysates were further purified and concentrated by PEG-precipitation (24 h-incubation at 4 °C with poly-ethylene glycol 8000 (PEG-8000) and sodium chloride at a final concentration of 10% *w/v* and 1 M, respectively) and subsequent 0.2 µm filtration, centrifugation (10,000× *g*, 30 min, 4 °C), and re-suspension in either sterile TYES-B or sterile SM buffer, as described by [27,38].

*F. columnare*-infecting lytic Myoviridae phages FCL-2 (isolated in 2008 in Finland, infection of hosts in genetic group G) [34] and FCOV-F27 (isolated in 2017 in Finland, infection of hosts in genetic group C) [33] were used in the experiments (phages were previously isolated from tank water in fish farms during columnaris outbreaks). FCL-2 has previously been shown effective against columnaris infections in rainbow trout [28]. To test the interaction with rainbow trout eggs (section B, experiment III), crude lysates of each phage were produced, as described earlier [28]. To test the efficiency of phages in preventing *F. columnare* replication on eggs, the phage FCL-2 was produced and purified by tangential flow filtration with diafiltration by PhageCosultants Ltd. Briefly, 300 mL of the crude lysate was loaded on the Millipore Labscale Tangential Flow Filtration (TFF) System with Pellicon^®^ XL Ultrafiltration Module Biomax^®^ 100 kDa, 0.005 m^2^. The lysate was diafiltrated by using ultrafiltration membranes (PES, 100 kDa pass) to completely remove or lower the concentration of salt, solvent, and metabolites by exchanging the volume of the lysate to 0,9% NaCl three times.

### 2.3. Rainbow Trout Eyed Eggs

**Figure 1:**
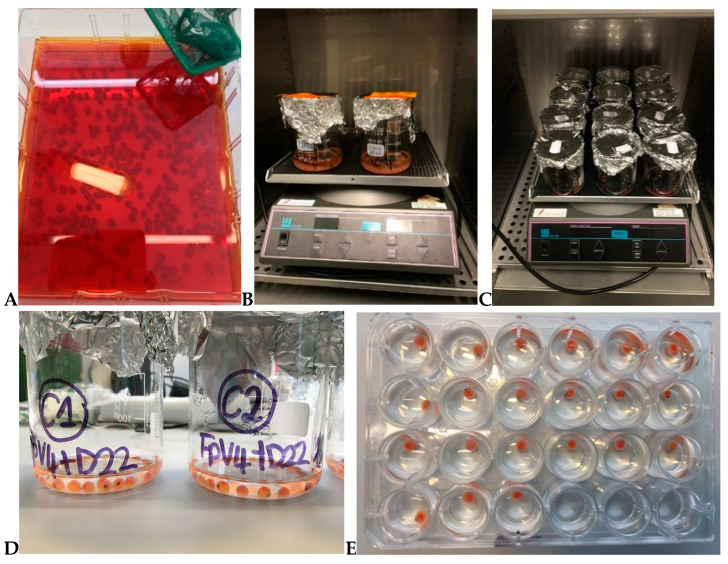
Illustrated overview of the experimental procedure followed in the various experiments concerning *F. psychrophilum* and its phages. (**A**) Disinfection of eyed eggs following standard procedures used in hatchery facilities (iodine-based solution) performed at the start of each experiment; (**B**) eyed eggs during the two-hour bacterial bath challenge with *F. psychrophilum* and incubation at 10 °C (experiments section A and C); (**C**) eyed eggs during the phage bath and incubation at 10 °C (experiments section B and C); (**D**) eyed eggs for phage bath placed in 250 mL sterile glass beakers (experiments section B and C); (**E**) eyed eggs during incubation in 24-well plates (experiments section A, B, and C) (photos by V.L. Donati).

Rainbow trout (*Oncorhynchus mykiss*) eyed eggs (>200 day degrees, dd) used for the experiments concerning *F. psychrophilum* and its phages were purchased from Troutex ApS (Egtved, Denmark). A few hours after arrival at the laboratory (Denmark), the experiments were performed. The status of the eyed eggs was inspected to reveal if any mortality had occurred during the transportation, whereafter the eggs were disinfected according to standard procedures performed at Danish rainbow trout hatcheries (10–15 min treatment in a iodine-based disinfectant for aquaculture) (100 ppm active iodine; 1% Actomar K30 (Desag AF, Uster, Switzerland)) [15] (Figure 1A). After disinfection, the eyed eggs were rinsed with sterile water before the bacteria and phage exposure experiments.

For the experiments concerning *F. columnare* and its phages, rainbow trout eyed eggs (>200 day degrees, dd) were received from a fish farm within a one-hour drive from the laboratory (Finland). The eggs were disinfected with the iodine-based disinfectant Buffodine^®^ (Evans Vanodine International plc, Lancashire, UK) at the farm according to the manufacturer’s instructions (10 min treatment), cold-transported to the lab, and used immediately in the experiments. Before the start of an experiment, six eggs were sampled for the presence of *F. columnare* and its phages and found negative.

### 2.4. Establishment of a Bath Bacterial Challenge Method (Section A)

A series of experiments was initially performed to establish a reproducible method to study the interactions of *F. psychrophilum* and rainbow trout eyed eggs at a small scale. These experiments were performed with the aim of (1) isolating the bacterium in connection with the eggs and (2) recording the effects of the bacterial challenge on the eggs’ survival during 24 h incubations. An additional experiment, focused on *F. psychrophilum* growth in different media, was performed. Furthermore, experiments targeting *F. columnare* were set up with the aim of evaluating the effects of temperature and medium on the eggs’ survival.

*F. psychrophilum* 950106-1/1 was chosen for the preliminary experiments. Disinfected eyed eggs were placed in 500 mL sterile glass beakers containing 200 mL of either bacterial solution (Exp. no. 1: 8.7 × 10^4^ CFU mL^−1^; Exp. no. 2: 1.5 × 10^7^ CFU mL^−1^; Exp. no. 3: 1.6 × 10^5^ CFU mL^−1^) or sterile TYES-B (control for the infection) and incubated for 2 h at 10 °C at 80–90 RPM (Figure 1B). After the bath challenge procedure, the eyed eggs were moved to sterile 24-well plates (one egg per well) (CELLSTAR^®^, Greiner Bio-One GmbH, Frickenhausen, Germany) containing 2 mL of sterile TYES-B (Exp. no. 1), sterile Milli-Q water (Exp. no. 2), or sterile SM buffer diluted 10 times in Milli-Q water (Exp. no. 3). Eyed eggs were transferred using sterile 10 µL inoculation loops (Figure 1E). The plates were covered with lids and incubated at 10 °C (at 80–90 RPM) for 24 h. In Exp. no. 1, three eggs were sampled at 1, 3, 21, and 25 h after the incubation in 24-well plates. In Exp. no. 2 and no. 3, three eggs were sampled right after the end of the bacterial bath (before the transfer to 24-well plates) and after 24 h of incubation. Exp. no. 1 was performed in December 2018, Exp. no. 2 in May 2019, and Exp. no. 3 in June 2019. Furthermore, to evaluate the growth of *F. psychrophilum* 950106-1/1 in Milli-Q water and 0.2 µm filtered tank water collected in our fish experimental facilities and compare to the growth in TYES medium, a growth experiment was performed as follows: 0.5 mL of a 72 h bacterial culture (5 mL) was transferred into either 100 mL of Milli-Q water, 100 mL of water from fish experimental facilities (fish tanks), or sterile TYES-B, and incubated at 15 °C. The experiment was performed in duplicates and the CFU count was performed at various time points.

In the case of *F. columnare*, various temperatures (5 °C (moved to 10 °C after 72 h), 15 °C, and 20 °C), in combination with different media (pre-aerated with pressurized air until 100% oxygen saturation, non-aerated sterile distilled water, or pre-aerated or non-aerated sterile Shieh medium) were tested (February 2019). For each group, 12 eyed eggs were placed in sterile 24-well plates (Nunc^TM^, Thermo Fisher Scientific, Rochester, USA) containing 2 mL of the selected medium and incubated at the settled temperature without any shaking (similarly as for *F. psychrophilum* in Figure 1E). Four of the 12 eyed eggs in each group were exposed to the *F. columnare* strain B480 by adding 10 μL of overnight culture (1.0 × 10^8^ CFU mL^−1^) directly to the wells, giving a final density of 5.0 × 10^5^ CFU mL^−1^. Survival of the eggs (embryo movement and blood flow observed under a light microscope) was followed in 24 h intervals for 144 h, except for the experiments performed at 20 °C, which were carried out until 72 h. In the case of bacterial exposure, samples from the media surrounding the eggs were collected from at least two wells per treatment at 24, 48, and 96 h.

### 2.5. Interactions of Phages with Rainbow Trout Eyed Eggs (Section B)

In this section, the effects of phages on rainbow trout eyed eggs’ survival in the absence of pathogens were evaluated. The experiments were also aimed at evaluating if phages could interact with the surface of the eggs. The effects of two selected *F. psychrophilum* bacteriophages (FpV4 and FPSV-D22; singularly) were tested by constant (Exp. I, section B) and by short-term bath exposure (Exp. II, section B). Similarly, the effects of two selected *F. columnare* bacteriophages (FCL-2 and FCOV-F27, singularly; Exp. III, section B) were tested. An overview of the experiments performed in this section is presented in Table 1.

#### 2.5.1. Constant Exposure of Eyed Eggs to *F*. *psychrophilum* Phages (Exp. I, Section B)

Eyed eggs were constantly exposed to phages FpV4 (3.0 × 10^5^ PFU mL^−1^ crude lysate and 1.0 × 10^6^ PFU mL^−1^ PEG-purified in TYES-B) and FPSV-D22 (1.2 × 10^7^ PFU mL^−1^ PEG-purified in TYES-B) for 144 h (the experiment was performed in April 2019). A control group without phage exposure was included (eggs were placed in sterile TYES-B). After disinfection, seventy-five eyed eggs were placed in 24-well plates using sterile 10 µL inoculation loops (all groups contained 23 eggs except the group where eggs were exposed to FpV4 in crude lysate where 16 eggs were incubated) with 2 mL of phage solution (sterile TYES broth for the control) (Figure 1E). Covered with lids, the plates were incubated at 10 °C at 80–90 RPM. After 2, 27, 49, and 71 h of incubation, three eggs and their correspondent well content per group were collected. At 144 h after the start of the experiment, the status of three eggs (alive/dead; hatched/not hatched) was characterized, and only the well content was collected for phage analysis.

#### 2.5.2. Bath Exposure of Eyed Eggs to *F*. *psychrophilum* Phages (Exp. II, Section B)

In this experiment (performed in April 2019), eighty-one rainbow trout eyed eggs were bathed for 4 h at 10 °C at 80–90 RPM either in phage solutions (1.9 × 10^7^ PFU mL^−1^ FpV4 or 8.2 × 10^7^ PFU mL^−1^ FPSV-D22 crude lysates) or in sterile TYES-B, for the control group. The bath procedures were performed in 250 mL sterile glass beakers containing 80 mL of phage or control solution (27 eggs for each treatment) (Figure 1C). After phage exposure, eggs were subdivided into 24-well plates (24 eggs per group) with 2 mL sterile Milli-Q water (one egg per well) using sterile 10 µL inoculation loops. Plates were covered and incubated at 10 °C at 80–90 RPM for 144 h (Figure 1E). At 0 h and 24, 46, and 68 h after the end of phage bath exposure, three eggs and their correspondent well content per group were collected for further phage analysis. At 144 h, the status of three eggs (alive/dead; hatched/not hatched) was characterized, and only the well content was sampled for phage analysis.

#### 2.5.3. Bath and Constant Exposure of Eyed Eggs to *F*. *columnare* Phages (Exp. III, Section B)

Eyed eggs were exposed to phages FCL-2 or FCO-F27 (1.0 × 10^9^ PFU mL^−1^; crude lysates) diluted in either sterile distilled water or in Shieh medium at 10 °C. Phage exposure was performed by either a 30 min bath in a Petri dish (40–50 mm Ø, 15 mL medium volume, at 60 RPM) or constant exposure in 24-well plates (no shaking). Eggs without phage treatment and a phage lysate without eggs served as controls. For constant exposure and after the phage bath, eggs (8 per group) were individually placed in 24-well plates containing 2 mL of either sterile distilled water or Shieh medium (similarly as for *F. psychrophilum* in Figure 1E). Bathed eggs were moved into wells with only medium (distilled water or Shieh medium). Eyed eggs for constant exposure experiments were moved directly to wells containing the phages. Eyed eggs were moved using sterile disposable forceps. Survival of the eggs was determined at 0, 24, 48, and 96 h. Phage density was determined both from eggs and the corresponding well content at 0, 24, and 48 h.

### 2.6. Evaluation of Phages as Pathogen Control Agents (Section C)

In this section, the experiments were aimed at assessing the potential of phages as pathogen control agents (an overview is presented in Table 1). The effects of two selected *F. psychrophilum* phages (FpV4 and FPSV-D22; mixed 1:1) in controlling *F. psychrophilum* 950106-1/1 and the strain 160401-1/5N were tested by a 48 h bath exposure (Exp. I, section C). Similarly, the effects of the *F. columnare* phage FCL-2 in controlling *F. columnare* B185 were tested by either constant or bath phage exposures (Exp. II, section C).

#### 2.6.1. Phage Bath of *F*. *psychrophilum* Challenged Eggs (Exp. I, Section C)

**Figure A1:**
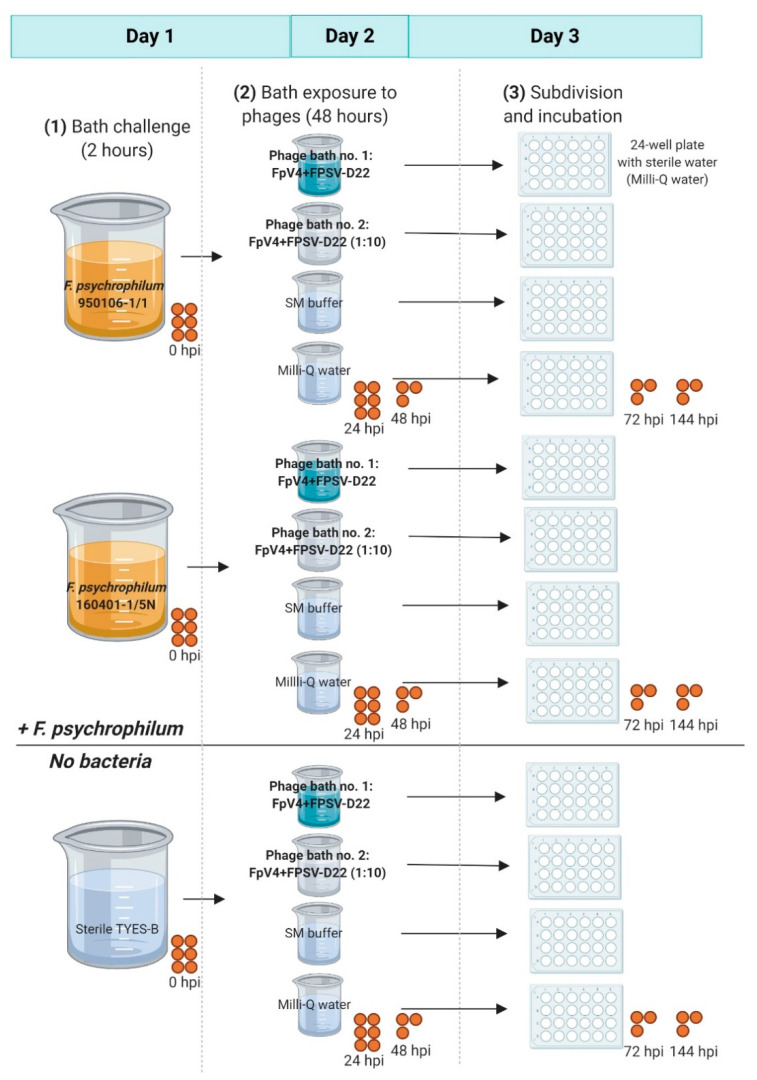
Exp. I section C: experimental set up. (**1**) Rainbow trout eyed eggs were bath-challenged for 2 h with either *F. psychrophilum* strain 950106-1/1 or the strain 160401-1/5N (2.0 × 10^6^ CFU mL^−1^), while control eggs were placed in sterile TYES-B. The procedure was performed in 600 mL sterile glass beakers containing 200 mL of the selected solution (135 eggs per beaker). (**2**) Eyed eggs were exposed to phages FpV4 and FPSV-D22 (mixed 1:1) for 48 h by bath: phage bath no. 1 (10^9^ PFU mL^−1^) and phage bath no. 2 (10^8^ PFU mL^−1^). Phage bath controls with SM buffer and Milli-Q water were included. The procedure was performed in 250 mL sterile glass beakers (30 eggs per beaker) containing the selected solution (20 mL). (**3**) Eyed eggs were divided in 24-well plates containing 2 mL of sterile Milli-Q water (one egg per well). For each step, eyed eggs were incubated at 10 °C at 80–90 RPM. Eyed eggs were sampled during the experiment as indicated by the round orange circles resembling trout eyed eggs. At 0 and 24 hpi, six eggs were sampled to compare the standard sampling procedure to the one including a drying step. hpi = hours post infection. Created with Biorender.com (the figure was exported under a paid license subscription).

*F. psychrophilum* bath-challenged eyed eggs were exposed to a two-component phage solution (phages FpV4 and FPSV-D22 mixed 1:1) for 48 h and then transferred to individual wells for the examination of phage and pathogen abundance (experimental set up presented in Figure A1). The experiment was performed in June 2020.

At first, eyed eggs were bath-challenged (2 h, 10 °C, 80–90 RPM) either with *F. psychrophilum* strain 950106-1/1 or the strain 160401-1/5N at a concentration of 2.0 × 10^6^ CFU mL^−1^. Control eggs were placed in sterile TYES-B. To perform the challenge, 135 disinfected eyed eggs were placed in 600 mL sterile glass beakers containing 200 mL of bacterial solution or sterile TYES-B (Figure 1B). Subsequently, eyed eggs were moved to 250 mL sterile glass beakers (30 eggs per beaker) containing either the selected phage solution (20 mL) or the phage bath controls (20 mL of sterile SM buffer or Milli-Q water) using sterile 10 µL inoculation loops (SARSTEDT AG & Co. KG, Nümbrecht, Germany) (one per group) (Figure 1C). For phage bath procedures, PEG-purified solutions of phage FpV4 and FPSV-D22 at a concentration of 3.9 × 10^8^ PFU mL^−1^ and 1.3 × 10^9^ PFU mL^−1^, respectively, were mixed 1:1 to a final concentration of 2.2 × 10^9^ ± 1.6 × 10^9^ PFU mL^−1^ (phage bath no. 1) and diluted 10 times in SM buffer for phage bath no. 2 (final concentration of 1.3 × 10^8^ ± 4.8 × 10^7^ PFU mL^−1^). The selected volume (20 mL) was considered enough to cover the eggs during the incubation (Figure 1D). After a 48 h incubation at 10 °C (at 80–90 RPM), eggs were divided in 24-well plates containing 2 mL of sterile Milli-Q water (one egg per well) with the help of sterile 10 µL inoculation loops (one per egg) (Figure 1E). Plates were covered with lids and incubated at 10 °C at 80–90 RPM.

Eyed eggs and the corresponding bath or well content were sampled for bacteria and/or phage quantification at the end of the bacterial challenge (0 h post infection, hpi), during phage exposure (24 and 48 hpi) and during the subsequent incubation in 24-well plates (72 and 144 hpi). For the sampling points 0 and 24 hpi, six eggs were sampled and, during the sampling procedure of three of them, an additional drying step was included. For the following sampling points, three eggs were collected and sampled without any drying step.

#### 2.6.2. Phage Exposure of *F*. *columnare* Challenged Eggs (Exp. II, Section C)

**Figure A2:**
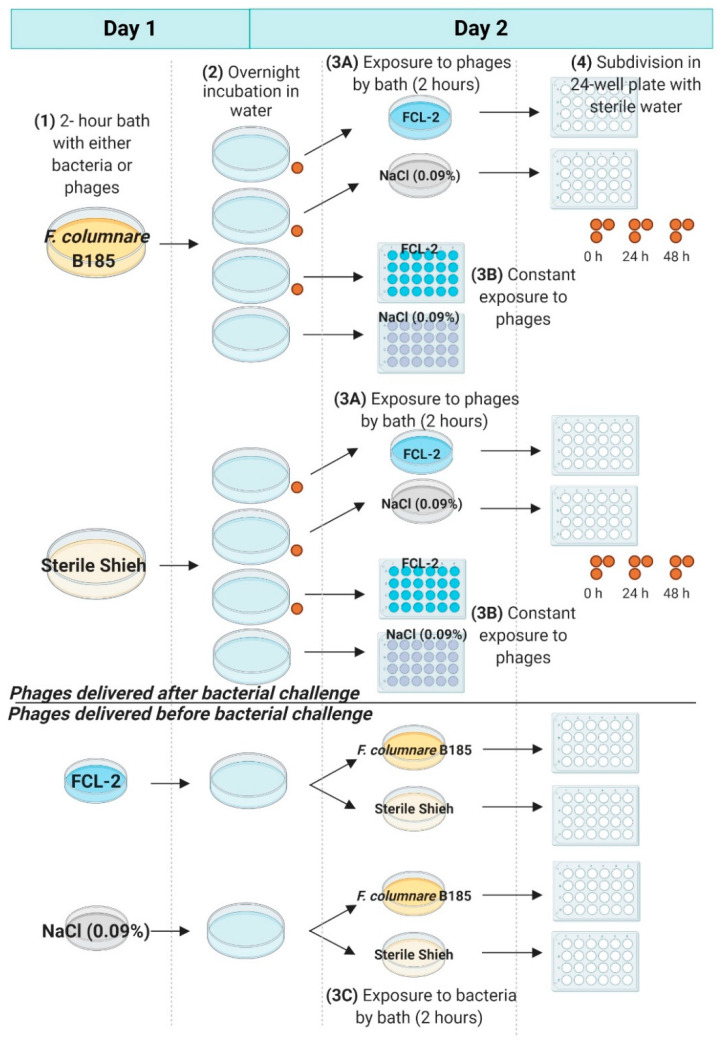
Exp. II section C: experimental set up. Rainbow trout eyed eggs were exposed to phages after (upper panel) or before bacterial challenge (lower panel). (**1**) Eyed eggs were bathed for 2 h with *F. columnare* B185 (5.0 × 10^6^ CFU mL^−1^) or sterile Shieh medium (upper panel) in 140 mm Ø Petri dishes (97–99 eggs per dish–100 mL volume), and with the phage FCL-2 (2.5 × 10^7^ PFU mL^−1^) or NaCl (0.09%) (lower panel) in 90 mm Ø Petri dishes (24 eggs per dish–35 mL volume). (**2**) Eggs were moved into 140 mm Ø Petri dishes (22–25 eggs per dish–100 mL volume) with water and incubated overnight. (**3**) Eyed eggs previously exposed to *F. columnare* were either (**3A**) bathed for 2 h or (**3B**) moved directly into 24-well plates in either FCL-2 phage solution (2.5 × 10^7^ PFU mL^−1^) or NaCl (0.09%) (24 eggs per group) (upper panel). (**3C**) Eyed eggs previously exposed to phages were bath-exposed to *F. columnare* strain B185 (5.0 × 10^6^ CFU mL^−1^) or sterile Shieh medium for 2 h (12 eggs per group) (lower panel). Bath exposures to either phages or bacteria were performed in 90 mm Ø Petri dishes (35 mL volume). (**4**) Following the 2 h bath, eyed eggs were transferred into 24-well plates containing 2 mL of water (one per well). Eyed eggs were sampled during the experiment as indicated by the round orange circles resembling trout eyed eggs. h = hours post phage exposure. Created with Biorender.com (the figure was exported under a paid license subscription).

*F. columnare* strain B185 and its phage FCL-2 (purified by diafiltration and diluted in NaCl 0.9%) were used in the experiment where eyed eggs were exposed to phages after (upper panel in Figure A2) or before the bacterial challenge (lower panel in Figure A2). Ion-exchanged water was used as a medium for the eggs, and the temperature was 10 °C.

At first, eyed eggs were bathed for 2 h (at 60 RPM): (a) with *F. columnare* B185 (5.0 × 10^6^ CFU mL^−1^) or with sterile Shieh medium (diluted in ion-exchange water in the same extent as done for the bacterium) in 140 mm diameter Petri dishes (SARSTEDT AG & Co. KG, Nümbrecht, Germany) (97–99 eggs per dish–100 mL volume), or else (b) with the phage FCL-2 (2.5 × 10^7^ PFU mL^−1^) or with NaCl (0.09%) in 90 mm diameter Petri dishes (24 eggs per dish–35 mL volume). After the baths, the eggs were moved with sterile forceps in 140 mm diameter Petri dishes (22–25 eggs per dish–100 mL volume) containing water and incubated overnight without agitation. In addition, 24 additional eggs (12 per each group) were placed directly in 24-well plates containing water or NaCl (0.09%) without any preliminary bath procedure and observed constantly during the experiment. After the overnight incubation, the viability of all the eggs was checked and, in the bath bacterial challenge groups, 3 eggs and their corresponding well content (per treatment: + or − *F. columnare*) were sampled to quantify the bacterial densities and the phage titers.

Following the overnight incubation, eyed eggs previously exposed to *F. columnare* were either bathed for 2 h or moved directly into 24-well plates in either FCL-2 phage solution (2.5 × 10^7^ PFU mL^−1^) or NaCl (0.09%) (24 eggs per group). Eyed eggs previously exposed to phages were bath-exposed to *F. columnare* strain B185 (5.0 × 10^6^ CFU mL^−1^) or sterile Shieh medium for 2 h (12 eggs per group). Bath exposures to either phages or bacteria were performed in 90 mm diameter Petri dishes (35 mL volume) at 60 RPM. Following the 2 h bath, eyed eggs were transferred to 24-well plates containing 2 mL of water (one per well). In the part of the experiment where eyed eggs were at first exposed to the bacterium and then to the phages (constantly or by bath), the viability of the eggs was observed immediately after transferring the eggs to 24-well plates and then at 24 h intervals until 144 h. In addition, three eggs and their corresponding well content were sampled at 0, 24, and 48 h to quantify the bacterial densities and the phage titers.

### 2.7. Eyed Eggs Sampling Procedure

**Figure 2:**
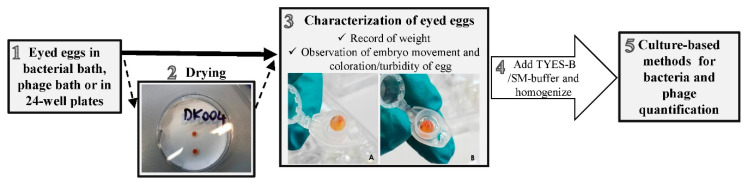
Graphical flow of the eyed eggs sampling procedure in relation to the experiments focused on *F. psychrophilum* and its phages. (**1**) Eyed eggs placed in the bacterial bath, the phage bath, or in 24-well plates were sampled at the selected time points. (**2**) A drying step was included for a selected number of eggs in Exp. I in section C. (**3**) Eyed eggs were characterized (A: example of turbid egg; B: example of normal coloration). (**4**) Sampled eggs were processed and homogenized. (**5**) According to the scope of the experiment, bacteria were enumerated and samples for phage analysis stored.

The graphical overview in Figure 2 refers to the eyed eggs sampling procedure followed in the experiments concerning *F. psychrophilum* and its phages. Additional information in relation to *F. columnare* are presented at the end of this paragraph.

The sampling procedure was developed based on the previous work of [39,40,41]. Eyed eggs placed in the bacterial bath, the phage bath, or in 24-well plates (Figure 2 step 1) were collected at the selected time points using a sterile 10 µL inoculation loop (SARSTEDT AG & Co. KG, Nümbrecht, Germany) and placed in pre-weighted sterile 1.5 mL micro tubes (SARSTEDT AG & Co. KG, Nümbrecht, Germany) (Figure 2 step 3). For the Exp. I in section C, a drying step was included for a selected number of eggs (Figure 2 step 2), which were placed on sterile filter paper for a few seconds (Whatman^®^ cat. no. 1003 090, Cytiva, Marlborough, USA) and then transferred to sterile 1.5 mL micro tubes. The weight was recorded and sampled eggs were characterized by observing the embryo movement and by recording the coloration/presence of turbidity of the egg (Figure 2 step 3). Dead eggs were identified by a whitish/opaque coloration, as previously described [40]. Sampled eggs were then cut and fragmented with the use of sterile scissors, and a fixed volume of TYES-B (experiments section A and Exp. II section C) or SM buffer (Exp. I and II section B) was added according to the scope of the experiment. Samples were thereafter homogenized by vortexing (15–20 s) (Figure 2 step 4). Finally, bacteria were enumerated by CFU counts, and the homogenized content was stored for subsequent phage quantification (Figure 2 step 5).

During Exp. I and II in section B, where our aim was to quantify *F. psychrophilum* phages in connection with the eyed eggs over time, 300 µL of sterile SM buffer was added to the sampled eggs (Figure 2 step 4), and after the homogenization procedure, 5 µL of chloroform was added and samples were stored for further phage analysis. For each sampled egg, the corresponding well content was also collected for phage analysis (300 µL of well content was placed into sterile 1.5 mL micro tubes and 5 µL of chloroform was added). The well content was streaked on TYES-A and Blood-A plates to assess the growth of bacteria/fungi. TYES-A plates were incubated at 15 °C and Blood-A plates at 20 °C for 4–5 days.

During the experiments of section A and C (concerning *F. psychrophilum)*, eyed eggs were sampled to quantify solely the bacterium (section A) or both the bacterium and the phages (Exp. I section C) in connection with the eggs. In this case, after egg status characterization (Figure 2 step 3), a fixed volume of sterile TYES-B (300–1000 µL in experiments of section A; 700 µL in Exp. I section C) was added to each egg sample and homogenized (Figure 2 step 4). Ten-fold serial dilutions were immediately performed and spread on TYES-A plates in order to estimate the bacterial concentration by CFU counts. Sampled eggs from the bacterial control groups (not exposed to *F. psychrophilum*) were also plated on TYES-A (no dilutions). For Exp. I section C, 300 µL of the homogenized egg samples was transferred into new sterile 1.5 mL micro tubes (SARSTEDT AG & Co. KG, Nümbrecht, Germany) and 5 µL of chloroform was added for subsequent phage analysis (Figure 2 step 5). In addition, phages and bacteria were also quantified in the corresponding well or bath content of each sampled egg. The concentration of bacteria was determined performing ten-fold serial dilutions of the well/bath content directly from the 24-well plate or the beaker used for bath procedures and plated on TYES-A plates. Bath/well content of sampled eggs from the bacterial control groups (not exposed to *F. psychrophilum*) were also plated on TYES-A (no dilutions). TYES-A plates were incubated at 15 °C for 4–5 days and CFU per mL of solution was estimated. For the Exp. I section C, 300 µL of the well/bath content was also placed in new sterile 1.5 mL micro tubes, 5 µL of chloroform was added, and the samples were stored at 5 °C in the dark for subsequent phage quantification. Homogenized eggs and the corresponding bath/well content were streaked on Blood-A to assess the growth of other bacteria/fungi and plates were incubated as mentioned earlier (Exp. I section C; only the well content for experiments of section A). The growth of bacteria other than *F. psychrophilum* on TYES-A plates was recorded (section A and C).

In the experiments concerning *F. columnare*, the survival of the eyed eggs was followed by observing the embryo movement and, in the experiments in section A, by observing the blood flow by a light microscope. In addition, and as performed for *F. psychrophilum*, samples for bacterium and phage detection/quantification were collected according to the scope of the experiment. In section A, the well content was streaked on Shieh agar plates, incubated at room temperature for 2 days, and the growth of *F. columnare* colonies recorded. In the Exp. II section B, the egg samples were processed similarly as for *F. psychrophilum*. Briefly, eyed eggs were placed in pre-weighted 1.5 mL Eppendorf tubes and crushed using a Bio Plas homogenization pestle (Thomas Scientific, Swedesboro, NJ, USA). A specific volume of Shieh medium was added (1:10 weight per volume) and the sample was mixed and centrifuged briefly to separate the supernatant, which was stored with chloroform for further phage quantification. For each sampled egg, the corresponding well content was also collected for phage analysis (300 µL media samples were stored at 4–6 °C with 1% chloroform). Finally, in the Exp. II section C, the eggs were not crushed, but were individually vortexed for 10 s in 400 µL of Milli-Q water, of which 100 µL was used to detect *F. columnare* (ten-fold dilutions plated on Shieh agar plates containing 1 µg mL^−1^ of tobramycin), and 150 μL was stored with chloroform for phage titration.

MALDI-TOF MS (Bruker Daltonic GmbH, Bremen, Germany) was used to confirm that the re-isolated bacteria were *F. psychrophilum* in doubtful cases, and to identify some of the background bacteria (if present) [42].

### 2.8. Detection and Quantification of Bacteriophages

Bacteriophage detection for phages infecting *F. psychrophilum* was performed as described by [24,25]. Egg and well content samples were centrifuged for 10 s at 10,000 RPM at 5 °C to separate chloroform at the bottom of the tube, and a phage spot method was performed [43]. Four milliliters of TYES soft agar (0.4% agar) mixed with 300 µL of a 48 h old *F. psychrophilum* broth culture (in exponential phase) was poured into a TYES-A plate [25,36]. Undiluted samples were then spotted in duplicate (section B) or triplicate (section C) (5 µL) on a bacterial lawn and incubated at 15 °C for 3–4 days. Phages were quantified by counting the plaques in individual spots. In the case of confluent or semi-confluent clearing areas, samples were diluted 10-fold (180 µL of SM buffer and 20 µL of sample) in triplicates and re-spotted on a bacterial lawn as described above.

Bacteriophage quantification for phages infecting *F. columnare* was performed as previously described by [28]. Three hundred microliters of an overnight-grown *F. columnare* was mixed with 3 mL of melted Shieh soft agar (0.7%) tempered to 47 °C, and poured on Shieh agar plates. Two microliters of the ten-fold dilutions of the phage samples (in sterile Milli-Q water) was spotted on top of the soft agar. Plaques were recorded after incubation for 2 days at room temperature.

### 2.9. Statistics

Statistical significant differences in the bacterial and phage concentrations were tested with GraphPad Prism version 8.4.0 for Windows, (GraphPad Software, San Diego, CA, USA, www.graphpad.com). For meaningful comparisons of two groups, values were compared with a two-tailed unpaired t-test. For comparison of three or more groups, values were compared with ANOVA. *p*-values for multiple comparisons were adjusted for Dunnet correction (adjusted *p*). *p*-values (*p*) below 0.05 were considered significant.

## 3. Results

### 3.1. Establishment of a Bath Bacterial Challenge Method (Section A)

**Figure 3:**
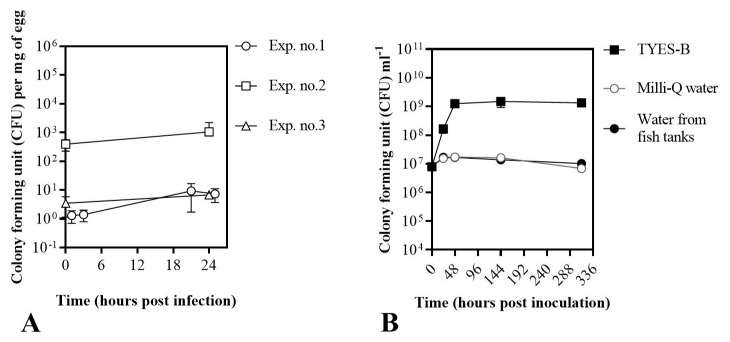
*F. psychrophilum* in connection with the eyed eggs in three independent experiments (section A) (**A**) and *F. psychrophilum* growth in Milli-Q and filter-sterilized water from fish tanks in comparison to TYES-B (**B**). In (**A**), values represent the mean and standard deviation of three biological replicates except in exp. no. 3 at 24 h post infection (*n* = 2). Control eyed eggs (bathed with sterile TYES-B) were negative to the bacteria for each experiment. In (**B**), values represent the mean and standard deviation of two replicates.

In the first part of our study, we developed an infection bath challenge method for rainbow trout eyed eggs, focusing on *F. psychrophilum*, with the aim of evaluating fish eggs’ survival in the established set up, and the bacterial growth and stability associated with fish eggs and different media (Figure 3, Table A1 and Table A2). In addition, since the optimum temperature for *F. columnare* is between 22–29 °C (depending on the strain) [44], while rainbow trout eyed eggs are normally incubated between 6 and 12 °C [45], the effects of different temperatures on the eggs’ survival were at first evaluated, also in combination with different media (Figure S1).

In the case of *F. psychrophilum*, all sampled eggs were characterized as alive based on movement and turbidity indicators (Table A1), and were subsequently recorded to be alive up to 6 days after the start of the experiments (data not shown). *F. psychrophilum* concentrations in connection with the eyed eggs correlated with the initial bacterial concentration of the bath (Figure 3A). After the bath challenge with 8.7 × 10^4^ CFU mL^−1^ (Exp. no. 1, 1 h post infection or hpi) and 1.6 × 10^5^ CFU mL^−1^ (Exp. no. 3, 0 hpi), the concentration of *F. psychrophilum* detected in connection with the eyed eggs was 1.3 ± 0.6 and 3.5 ± 2.4 CFU mg^−1^ of egg, respectively. When the eyed eggs were bathed in a higher concentration of bacteria (Exp. no. 2: 1.5 × 10^7^ CFU mL^−1^), the bacterial concentration on the eggs had increased to 3.9 × 10^2^ ± 1.7 × 10^2^ CFU mg^−1^ of egg (0 hpi). The concentration of bacteria detected in connection with eyed eggs was maintained within 24 h in the 24-well plates. The detection of bacteria other than *F. psychrophilum* was recorded, and is presented in Table A2. In additional independent experiments, we observed the growth of *F. psychrophilum* in Milli-Q and filter-sterilized tank water from fish stables (Figure 3B): the bacteria were not able to actively grow under these conditions, but they remained viable for the tested time frame (15 days)**.**

In the experiments concerning *F. columnare* (Figure S1), rainbow trout eyed eggs did not survive at 20 °C, and all movement was lost after 24 h in all the treatments (at 20 °C). Fish eggs were characterized as alive until 96–144 h when placed in water at 5 and 15 °C. The presence of nutrients (Shieh medium) reduced the time of egg survival. When the eggs were spiked with *F. columnare*, their survival was not affected and the bacteria could be isolated in the eggs incubated at 15 and 20 °C up to at least 48 hpi. At 5 °C, *F. columnare* could be isolated only at 24 hpi. Based on these results, the subsequent experiments concerning *F. columnare* and its phages were performed at 10 °C.

### 3.2. Interactions of Phages with Rainbow Trout Eyed Eggs (Section B)

#### 3.2.1. Constant Exposure of Eyed Eggs to *F*. *psychrophilum* Phages (Exp. I, Section B)

The tested phages did not seem to negatively affect the eggs’ survival (Table 2A). Sampled eggs were characterized as alive up to 49 and 71 h in all groups (only one egg out of 3 exposed to FpV4 in crude lysate was dead at 71 h). However, the embryo movement was not observed for a higher number of eggs exposed to the crude lysate compared to the other groups. In addition, at the termination of the experiment (144 h), most of the eggs in the sampled wells were dead except for two out of three in the PEG-purified FpV4 solution (hatched and alive) and one in the control group (not hatched and alive). Phages were diluted in sterile TYES-B, and this could have stimulated the growth of other bacteria/fungi (Table A3).

**Figure 4:**
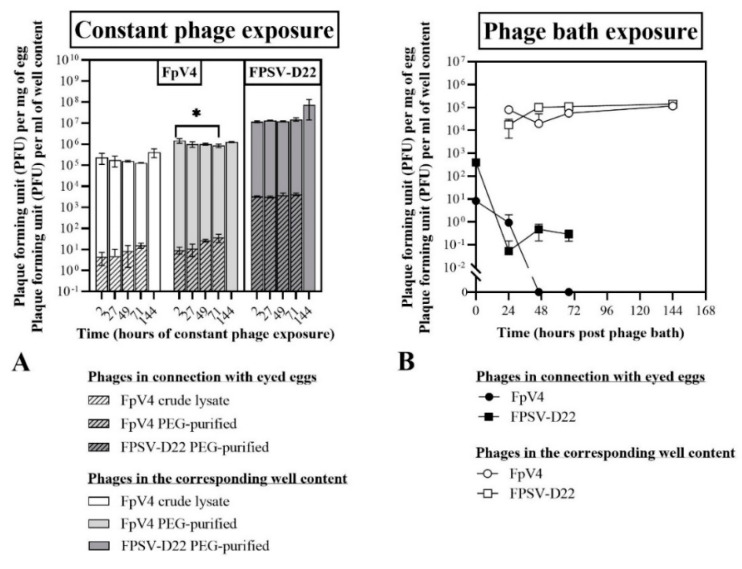
Exp. I and II, section B: phages associated with the eyed eggs and in the corresponding well content (**A**) during constant exposure to phage FpV4 (crude lysate and PEG-purified solutions) and FPSV-D22 (PEG-purified solution) and (**B**) after a 4 h bath exposure to phage FpV4 and FPSV-D22 (1.9 × 10^7^ PFU mL^−1^ FpV4 or 8.2 × 10^7^ PFU mL^−1^ FPSV-D22; crude lysates). Values represent the mean and standard deviation of three biological replicates. At 144 h, phages were quantified only for the well content. In **A**, * = statistically significant differences between the concentration of phages detected at 2 and 71 h in connection with eyed eggs (adjusted *p* = 0.0184) and in the corresponding well content (adjusted *p* = 0.0256). No other statistically significant differences were detected between phage concentrations within each group (**A**).

Two hours post constant phage exposure (Figure 4A), phages FpV4 and FPSV-D22 were detected in connection with the eyed eggs at a concentration of 4.4 ± 2.7 PFU mg^−1^ (FpV4 in crude lysate), 9.2 ± 3.3 PFU mg^−1^ (PEG-purified FpV4) and 3.2 × 10^3^ ± 2.2 × 10^2^ PFU mg^−1^ (PEG-purified FPSV-D22). The concentration of phages in connection with the eggs and in the corresponding wells was maintained over time in the groups with one exception (Figure 4A): the concentration of FpV4 associated with the eggs in the PEG-purified solution increased over time (adjusted *p* = 0.0184). No phages were detected in the control group.

To summarize, we observed that the tested phages did not seem to negatively affect the eyed eggs’ survival, and that the concentration of phage FpV4 in connection with the eyed eggs increased over time.

#### 3.2.2. Bath Exposure of Eyed Eggs to *F*. *psychrophilum* Phages (Exp. II, Section B)

Similarly to what was observed during constant phage exposure experiments (Exp. I section B), the survival of eyed eggs was not negatively affected when the eggs were bathed with either FpV4 (1.9 × 10^7^ PFU mL^−1^) or FPSV-D22 (8.2 × 10^7^ PFU mL^−1^) in crude lysates for four hours and then transferred to 24-well plates with sterile Milli-Q water (Table 2B). However, eggs were alive in all groups until the end of the experiment except one in the FPSV-D22 group at 144 h. Bacterial/fungal growth associated with the well content was detected firstly at 68 and 144 h in all three groups (Table A4).

The concentration of FpV4 and FPSV-D22 associated with the eyed eggs was 8.2 ± 0.7 PFU mg^−1^ of egg and 3.9 × 10^2^ ± 1.3 × 10^1^ PFU mg^−1^ of egg, respectively, at the end of the phage bath (Figure 4B). Subsequently, FpV4 phages were detected only after 24 h (0.9 ± 1.1 PFU mg^−1^ of egg) as no phages were detected in the following samplings. On the contrary, even if the concentration of FPSV-D22 phages in connection with the eggs dropped in the first 24 h (0.1 ± 0.1 PFU mg^−1^ of egg), it subsequently remained stable (46 h: 0.5 ± 0.3 PFU mg^−1^ of egg; 68 h: 0.3 ± 0.1 PFU mg^−1^ of egg). Bacteriophage FpV4 and FPSV-D22 maintained relatively constant concentrations in the well content of the sampled eggs, ranging from 8.0 × 10^4^ ± 1.3 × 10^4^ PFU mL^−1^ to 1.2 × 10^5^ ± 2.5 × 10^5^ PFU mL^−1^ (FpV4) and from 1.4 × 10^5^ ± 1.3 × 10^4^ PFU mL^−1^ to 1.7 × 10^4^ ± 1.3 × 10^4^ PFU mL^−1^ (FPSV-D22) during the 144 h incubation (Figure 4B).

To summarize, in this experiment, we observed that the survival of the eyed eggs was not affected by the phage bath (crude lysates) and that the concentration of phages in connection with the eyed eggs decreased over time. While FpV4 phages disappeared after 24 h, it was possible to detect FPSV-D22 phages until the last sampling (68 h).

#### 3.2.3. Bath and Constant Exposure of Eyed Eggs to *F*. *columnare* Phages (Exp. III, Section B)

The surrounding medium influenced the survival of the eggs (data not shown). While all eggs had died after 96 h incubation in Shieh medium, only 16.67% mortality was observed in water, independent of the presence of phages.

Phages could not be isolated from bath-treated eggs despite the high phage titers in the surrounding liquid (Table 3). Only a few eggs were positive to FCL-2 and FCOV-F27 with a concentration ≤ 10^2^ PFU egg^−1^. Both phages (FCL-2 and FCOV-F27) could be isolated from the corresponding well content (water/Shieh medium) from the bath, constant phage exposure, and phage control treatments at all the sampling points. The titers varied between 10^5^–10^9^ PFU mL^−1^ depending on phage, time point, and treatment (Table 3). Shortly, both phages had somewhat higher titers in Shieh medium than in water, FCL-2 had higher titers than FCOV-F27, and constant treatments had higher titers than bath treatments. However, phages did not seem to attach efficiently to the eggs.

### 3.3. Experiments to Evaluate the Use of Phages as Control Agents (Section C)

#### 3.3.1. Phage Bath of *F*. *psychrophilum* Challenged Eggs (Exp. I, Section C)

**Figure 5:**
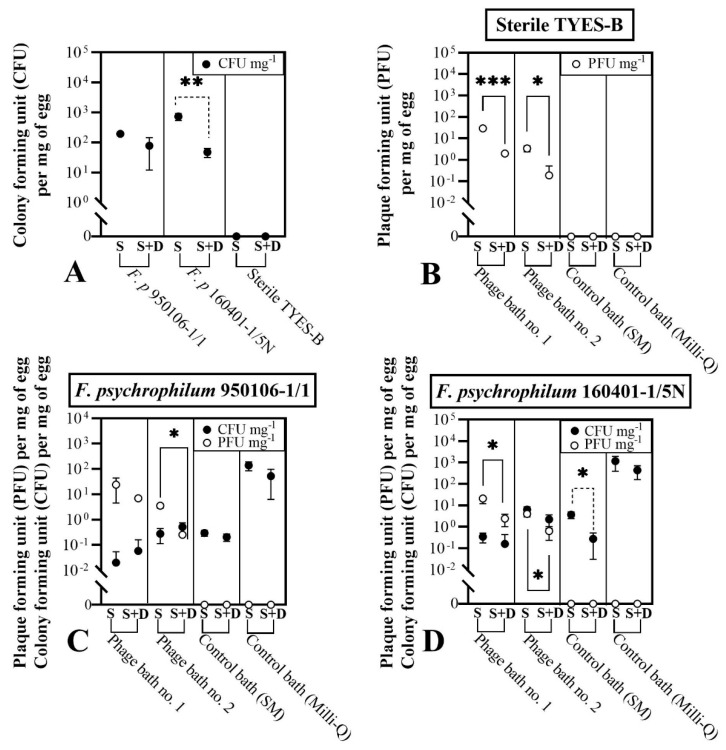
Exp. I, section C: effects of the drying procedure on bacterial and phage concentrations in connection with eyed eggs. Comparison between standard sampling (indicated by “S”) and sampling with the additional drying step (indicated by “S + D”) for eyed eggs sampled at 0 hpi (**A,** right after the bath challenge) and at 24 hpi (**B**–**D**) that were previously bath-challenged with TYES-B (control, **B**), *F. psychrophilum* 950106-1/1 (**C**) and *F. psychrophilum* 160401-1/5N (**D**). Values represent the mean and standard deviation of three biological replicates. Unpaired t tests of log-transformed values were performed. Statistically significant comparisons (solid lines for phage concentrations and broken lines for bacteria concentrations) are visualized as follows: *p* < 0.05 (*), *p* < 0.001 (**), *p* < 0.0001 (***). Phage bath no. 1: 10^9^ PFU mL^−1^; phage bath no. 2: 10^8^ PFU mL^−1^.

**Figure 6:**
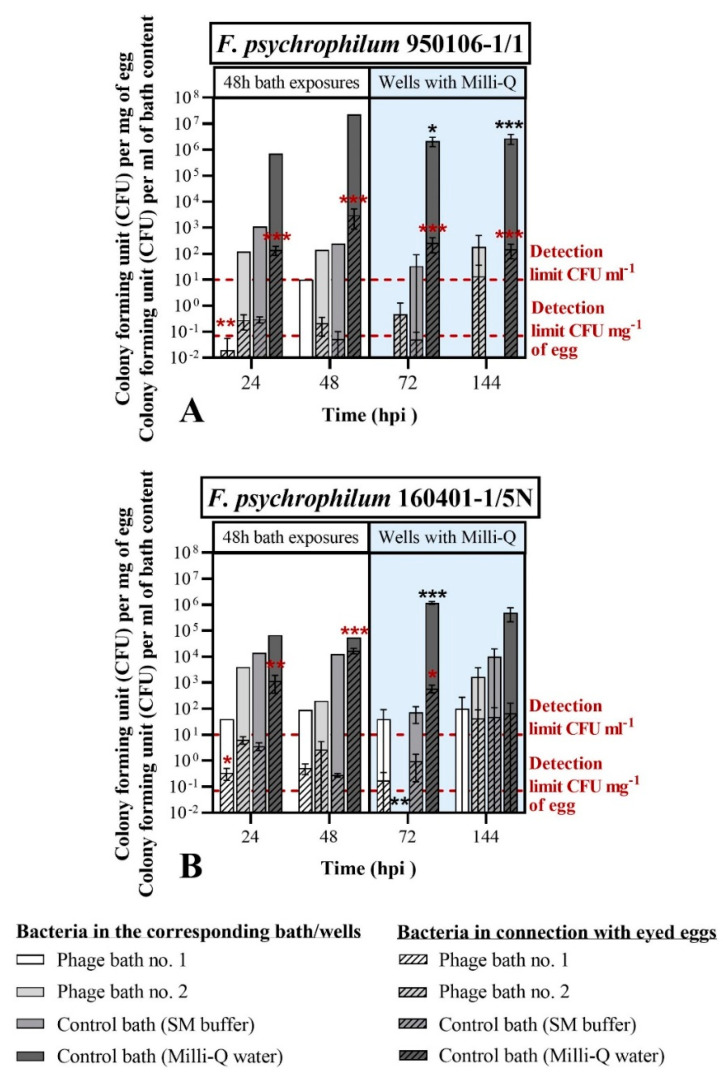
Exp. I, section C: *F. psychrophilum* 950106-1/1 (**A**) and *F. psychrophilum* 160401-1/5N (**B**) in connection with the eyed eggs and in the corresponding bath/well. After the bacterial challenge, eyed eggs were bath-exposed to phages FpV4 and FPSV-D22 mixed 1:1 (phage bath no. 1: 10^9^ PFU mL^−1^; phage bath no. 2: 10^8^ PFU mL^−1^; or control baths containing either SM buffer or Milli-Q water) for 48 h and subsequently moved to 24-well sterile plates containing sterile Milli-Q water (in light blue). Values represent the mean and standard deviation of three biological replicates except for the bath content at 24 and 48 hpi (*n* = 1). In the control group for bacterial infection (control bath with TYES-B), *F. psychrophilum* was not detected in eyed eggs and in the corresponding bath/wells. For the concentration of bacteria, the detection limit is indicated by red broken lines (calculated as 1 CFU was observed in the undiluted egg (mean weight = 100 mg) or well sample). Unpaired t tests of log-transformed values were performed to compare the tested conditions (phage baths and Milli-Q water-control bath) with the SM buffer-control bath. Statistically significant comparisons are visualized on top of each column (red: eyed egg values; black: well values) as follows: *p* < 0.05 (*), *p* < 0.001 (**), *p* = 0.0001 (***).

**Figure 7:**
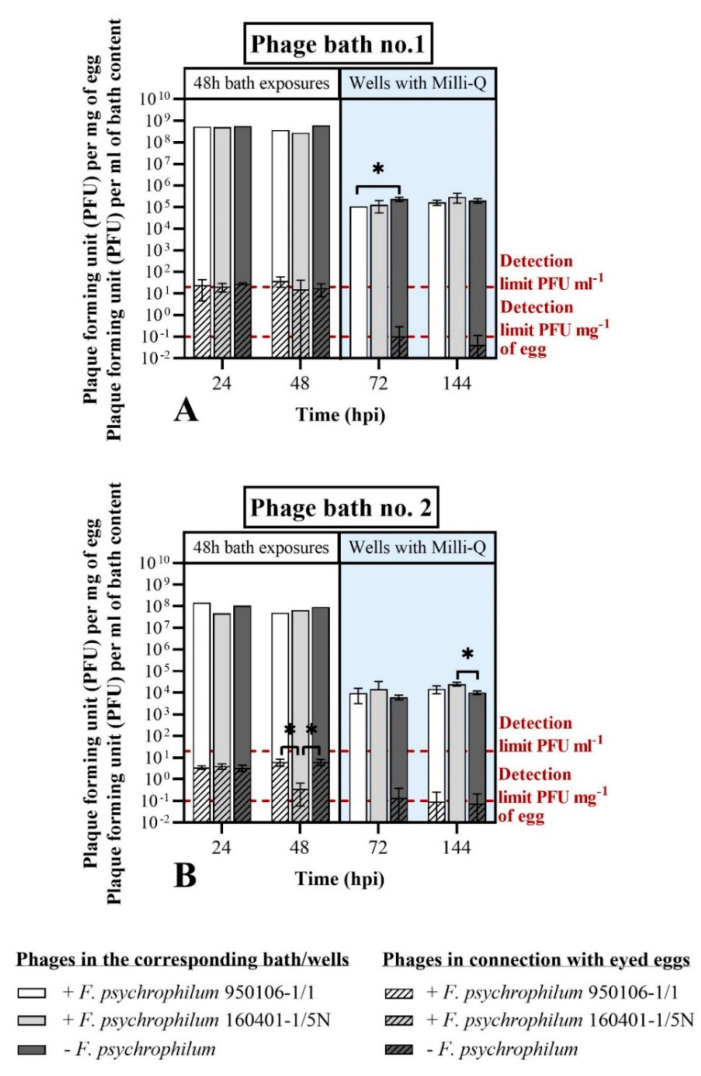
Exp. I, section C: phages FpV4 and FPSV-D22 in connection with the eyed eggs and in the corresponding bath/well following phage bath exposures: (**A**) phage bath no. 1 (10^9^ PFU mL^−1^) and (**B**) phage bath no. 2 (10^8^ PFU mL^−1^). In the phage bath control groups (containing either SM buffer or Milli-Q water), phages FpV4 and FPSV-D22 were not detected. After the bacterial challenge, eyed eggs were bath-exposed to phages FpV4 and FPSV-D22 for 48 h and subsequently moved to 24-well sterile plates containing sterile Milli-Q water (in light blue). Values represent the mean and standard deviation of three biological replicates, except for the bath content at 24 and 48 hpi (*n* = 1). For the concentration of phages, the detection limit is indicated by red broken lines (calculated as 1 PFU was observed in only one of the triplicate spots in the undiluted egg (mean weight = 100 mg) or well sample). Unpaired t tests of log-transformed values were performed to compare the tested conditions. Statistically significant comparisons are indicated as follows: *p* < 0.05 (*), *p* < 0.001 (**), *p* = 0.0001 (***). *F. psychrophilum* 950106-1/1 and the strain 160401-1/5N were used as bacterial hosts for the phage quantification analysis according to which strain was used in the bacterial bath challenge.

After the bacterial challenge with either *F. psychrophilum* 950106-1/1 or the strain 160401/1-5N (sterile TYES-B for the control), eyed eggs were bath-exposed to phages FpV4 and FPSV-D22 (mixed 1:1) for 48 h. Two control baths were included: one containing SM buffer (the buffer where the phages were purified in) and the other with Milli-Q water (to evaluate the effect of the buffer). Subsequently, eyed eggs were moved to 24-well sterile plates containing sterile Milli-Q water (experimental set up in Figure A1). The results of this experiment are presented in Figure 5, Figure 6 and Figure 7. The time point at which the bacterial bath challenge was finalized is named as 0 h post infection (hpi).

The first objective of this experiment was to study the association of phages and bacteria with the surface of the eyed eggs. Thus we compared the number of bacteria and phages per mg of egg at 0 and 24 hpi, sampled with either the standard procedure (S) or including a drying step (S + D), to assess to what extent the bacterial cells and the phages were firmly attached to the egg surface or associated with the liquid around the eggs (Figure 2 step 2). The results are presented in Figure 5. When the eyed eggs were bath-challenged with *F. psychrophilum* 950106-1/1, 1.9 × 10^2^ ± 3.2 × 10^1^ CFU mg^−1^ of egg was found using the standard procedure (S) at 0 hpi, and no significant loss of bacteria by the drying procedure was observed (S + D: 7.7 × 10^1^ ± 6.5 × 10^1^ CFU mg^−1^ of egg; *p* = 0.0902) (Figure 5A). This was also observed at 24 hpi (Figure 5C). The bacterial concentrations were 0.3 ± 0.1 and 1.4 × 10^2^ ± 5.4 × 10^1^ CFU mg^−1^ of egg in SM buffer and Milli-Q water, respectively, but with no significant difference between the S and S + D treatments. Exposure of the *F. psychrophilum* 950106-1/1-challenged eggs to phages did not influence the effects of the drying step on bacterial abundance.

The concentration of *F. psychrophilum* 160401-1/5N, on the other hand, seemed to be more affected by the inclusion of the drying step, as a 10-fold decrease after drying was detected at 0 hpi (S: 7.3 × 10^2^ ± 2.0 × 10^2^ CFU mg^−1^ of egg; S + D: 7.7 × 10^1^ ± 1.6 × 10^1^ CFU mg^−1^ of egg; *p* = 0.0004) (Figure 5A)**.** A similar effect was observed at 24 hpi when the eyed eggs were placed in SM buffer (S: 3.6 ± 1.2 CFU mg^−1^ of egg; S + D: 0.3 ± 0.2 CFU mg^−1^ of egg; *p* = 0.0056), but no significant changes were observed in the other groups (Figure 5D). Overall, these findings show that a fraction of the two selected *F. psychrophilum* strains was tightly attached to the eyed eggs’ surface and was not detached by the drying step. Additionally, there was a general decrease in egg-associated bacteria over 24 h incubations, even in the SM buffer control groups.

Phages seemed to be less closely attached to the surface of the eyed eggs. The inclusion of the drying step caused a 10- to a 100-fold decrease in phage concentrations in connection with the eyed eggs in each of the tested cases, independent of the presence of the bacteria (Figure 5B–D). For example, the number of phages recorded at 24 hpi in connection with the eyed eggs not exposed to *F. psychrophilum* (sterile TYES-B; Figure 5B) was 2.9 × 10^1^ ± 2.6 PFU mg^−1^ of egg for the S procedure compared to 0.7 ± 0.2 PFU mg^−1^ of egg for the S + D procedure (*p* < 0.0001) for eggs bathed in phage bath no. 1 (10^9^ PFU mL^−1^), and 1.4 ± 0.4 PFU mg^−1^ of egg for the S procedure compared to 0.1 ± 0.1 PFU mg^−1^ of egg for the S + D procedure (*p* < 0.05) for eggs bathed in phage bath no. 2 (10^8^ PFU mL^−1^).

**Figure A3:**
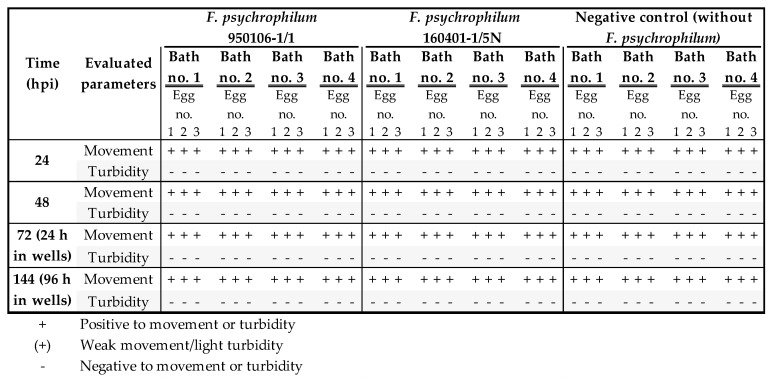
Exp. I section C: characteristics of sampled eggs. hpi = hours post infection. Bath no. 1: phage bath with of 10^9^ PFU mL^−1^; Bath no. 2: phage bath with 10^8^ PFU mL^−1^; Bath no. 3: control bath with SM buffer; Bath no. 4: control bath with Milli-Q water.

To evaluate the ability of phages to control *F. psychrophilum*, the bacterial and phage concentrations in connection with the eyed eggs were measured with the standard sampling procedure (S). Bacteria and phages were quantified on eggs sampled during the phage exposure in the bath treatment (at 24 and 48 hpi) and during the subsequent incubation in wells (at 72 and 144 hpi) (Figure 6 and Figure 7). No negative effect on the eyed eggs’ survival was observed in any of the groups, as all the eyed eggs sampled at 24, 48, 72, and 144 hpi were characterized as alive based on movement and turbidity indicators (Figure A3).

The concentration of bacteria per mg of egg was significantly reduced at 24 hpi in the case of phage bath exposure no. 1 (10^9^ PFU mL^−1^) in comparison to the control bath (SM buffer) (Figure 6). In fact, for bath-challenged eyed eggs with *F. psychrophilum* 950106-1/1, the concentration of bacteria associated with the eggs at 24 hpi was 0.02 ± 0.04 CFU mg^−1^ of egg in the phage bath exposure no. 1, compared with 0.3 ± 0.1 CFU mg^−1^ of egg in the case of the SM buffer-bath control (*p* < 0.001), corresponding to a 15-fold reduction in egg-associated bacteria due to the phage treatment (Figure 6A). A similar effect of phage exposure was observed for *F. psychrophilum* 160401-1/5N at 24 hpi, where egg-associated bacteria were reduced from 3.6± 1.2 CFU mg^−1^ of egg in the SM buffer-bath control to 0.3 ± 0.2 CFU mg^−1^ of egg in the phage bath exposure no. 1 (*p* = 0.0022, Figure 6B).

**Figure A4:**
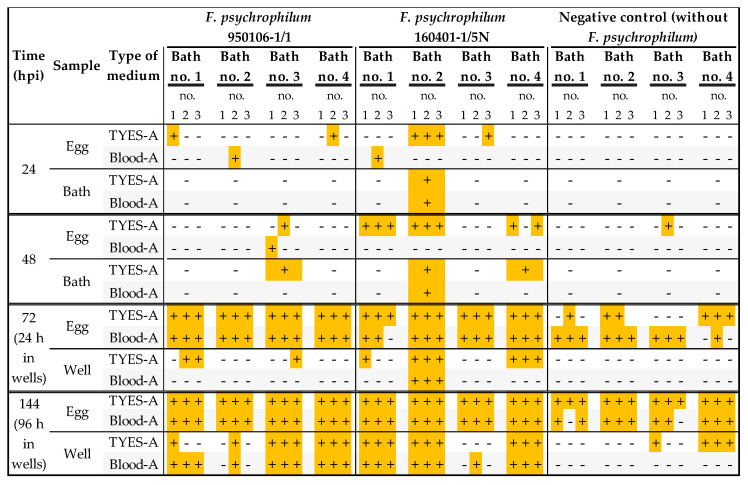
Exp. I section C: bacterial growth other than *F. psychrophilum* in sampled eyed eggs and the corresponding bath/wells was recorded. A plus symbol with an orange background indicates a positive detection of bacterial colonies in TYES-B and/or on Blood-A (“-” = no growth). Bath no. 1: phage bath with of 10^9^ PFU mL^−1^; Bath no. 2: phage bath with 10^8^ PFU mL^−1^; Bath no. 3: control bath with SM buffer; Bath no. 4: control bath with Milli-Q water.

Additionally, phage exposure reduced the bacterial abundance at 24 hpi in the bath content for both bacteria (Figure 6A,B). For *F. psychrophilum* 950106-1/1 no bacteria was detected in the phage bath no. 1 at 24 hpi, whereas 1.1 × 10^3^ CFU mL^−1^ (*n* = 1) were present in the SM buffer-bath control (Figure 6A). Similarly, the abundance of strain 160401-1/5N was reduced from 1.4×10^4^ CFU mL^−1^ in the SM buffer-bath control to 40.0 CFU mL^−1^ in phage bath no. 1 (*n* = 1, Figure 6B). These findings support the ability of FpV4 and FPSV-D22 to reduce the *F. psychrophilum* abundance at 24 hpi both on the egg surface and in the surrounding water. However, this effect of phage exposure was only temporary, as no significant difference between the bacterial abundances of the phage baths and the SM buffer-bath controls were observed at the following time points (48, 72, and 144 hpi). In addition, the growth and stability of the bacteria seemed to be increasingly negatively affected by incubation in the SM buffer-control bath compared to the Milli-Q water-control bath over time. As an example, at 72 hpi, the bacterial abundances detected in connection with the eyed eggs was 0.1 ± 0.1 and 2.6 × 10^2^ ± 1.5 × 10^2^ CFU mg^−1^ of egg for eyed eggs previously bathed in SM buffer and Milli-Q water, respectively, (*p* < 0.0001) (bath-challenged eyed eggs with *F. psychrophilum* 950106-1/1). The bacterial concentration in the corresponding wells containing the eggs was also significantly decreased (SM buffer bath-control: 3.3 × 10^1^ ± 5.8 × 10^1^ CFU mL^−1^; Milli-Q water-control bath: 2.1 × 10^6^ ± 8.4 × 10^5^ CFU mL^−1^; *p* < 0.05). A similar trend was observed for bath-challenged eyed eggs with *F. psychrophilum* 160401-1/5N at 72 hpi (bacteria associated with eyed eggs: 1.0 ± 0.8 CFU mg^−1^ of egg in the SM buffer bath control and 6.0 × 10^2^ ± 2.0 × 10^2^ CFU mg^−1^ of egg in the Milli-Q water control bath—*p* = 0.0027). This was also the case in the corresponding wells where 7.3 × 10^1^ ± 4.6 × 10^1^ CFU mL^−1^ were found in the SM buffer bath control compared to 1.2 × 10^6^ ± 1.4 × 10^5^ CFU mL^−1^ in the Milli-Q water control bath (*p* < 0.0001). The detection of bacteria/fungi other than *F. psychrophilum* was observed during the CFU enumeration and recorded (Figure A4).

The concentration of the two bacterial strains associated with the bath-challenged eyed eggs at 24 and 48 hpi in the Milli-Q water-control bath varied significantly with *F. psychrophilum* 950106-1/1, occurring in 10-fold lower numbers (1.4 × 10^2^ ± 5.4 × 10^1^ and 3.1 × 10^3^ ± 2.2 × 10^3^ CFU mg^−1^ of egg at 24 hpi and 48 hpi, respectively) than strain 160401-1/5N (1.2 × 10^3^ ± 7.8 × 10^2^ and 1.7 × 10^4^ ± 4.0 × 10^3^ CFU mg^−1^ of egg at 24 and 48 hpi, respectively) (24 hpi: *p* = 0.0030; 48 hpi: *p* = 0.0080), suggesting different adherence properties of the two strains (Figure 6).

As previously observed and mentioned in this results section (Figure 5), the phages FpV4 and FPSV-D22 did not seem to tightly connect with surface of the eyed eggs in this experiment. Even if the concentration of phages in connection with the eyed eggs was ~10^1^ PFU mg^−1^ (Phage bath no. 1) and ~10^0^ PFU mg^−1^ (phage bath no. 2) during the 48 h phage bath, very few eggs were positive to phages in the next sampling points (72 and 144 hpi) (Figure 7). However, FpV4 and FPSV-D22 were constantly detected over time, and their concentration was maintained in the baths and the wells, independent of the presence of the bacteria.

To summarize, the findings of this experiment showed that the two selected *F. psychrophilum* strains closely interact with the eyed eggs’ surface, but with different efficiencies. Furthermore, exposure of the challenged eggs to phages showed a 12- to 15-fold reduction in egg-associated bacteria for 24 h. However, the growth and stability of the bacteria were negatively affected in the SM buffer bath at all the time points, and the controlling effects of phages on the egg-associated bacteria were not maintained beyond 24 h.

#### 3.3.2. Phage Exposure of *F*. *columnare* Challenged Eggs (Exp. II, Section C)

The effects of phages on bacteria associated with eggs and their immediate proximity was also assessed with *F. columnare* (experimental set up in Figure A2). As for *F. psychrophilum*, most of the eggs survived until the end of the experiments (Figure S2). Some eggs hatched during the experiment.

In contrast to the experiments in section A (Figure S1), *F. columnare* was not isolated from any of the medium or egg samples taken at any sampling time point. It can thus be inferred that there was no growing or infective *F. columnare* in the treatments during the experiment, probably since the experiment was conducted at 10 °C. However, colonies of other environmental bacteria were observed (data not shown).

**Figure 8:**
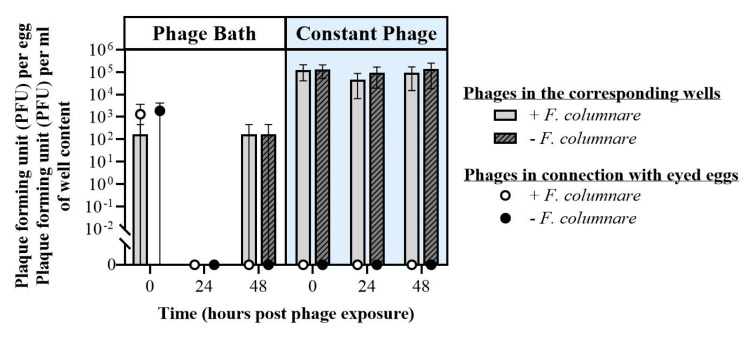
Exp. II, section C: phage FCL-2 in connection with the eyed eggs and in the corresponding wells following phage exposures (bath and constant) with and without bacterial challenge. In the phage control groups (sterile NaCl 0.09%; bath and constant experiments), phage FCL-2 was detected neither in the wells nor in the egg samples. Values represent the mean and standard deviation of three biological replicates. Unpaired t tests of log-transformed values were performed to compare the observed phage titers in the wells with and without bacterial exposure (constant phage exposure). No statistically significant difference was detected (*p* > 0.05).

Phages were only isolated from egg samples in low titers right after phage bath exposure experiment (Figure 8). One should notice that, in this experiment, eggs were not homogenized as in the previous experiments, but only vortexed in a fixed amount of water, which was then used for the phage and bacterial quantifications. Phages were isolated from well samples (*n* = 3) during constant phage exposure at all sampling points with a stable concentration, independent of the presence of *F. columnare* (e.g., 48 h in the case of the bacterial challenge: 9.3 × 10^4^ ± 7.8 × 10^4^ PFU mL^−1^). After the phage bath, it was possible to detect phages at 0 and 48 h in only one of the three wells.

## 4. Discussion

Our study aimed to evaluate the interactions between *Flavobacterium* spp. and rainbow trout eyed eggs and the potential of phages as control agents for these pathogens.

### 4.1. Experimental Infection Method

Although distant from the hatchery environment, the developed experimental set up allows for the study of bacterial and phage interactions with eyed eggs at a small scale under controlled conditions, as well as the production of reproducible results, meaning that the experimental set up might also be applied for other pathogenic bacteria.

No evident negative effects on survival were detected when eyed eggs were exposed to *F. psychrophilum* in our experiments (Section A and C), supporting previous findings of [46], in which no egg mortality was observed prior to hatching in the bacteria-challenged rainbow trout eyed eggs. However, the mortality of post-swim up fry exposed to *F. psychrophilum* was significantly higher than the controls in that study [46]. A different infection method was chosen by Ekman et al. [47], where the nano-injection of *F. psychrophilum* into the yolk of fertilized rainbow trout eggs was performed with the aim of mimicking the vertical transmission of this pathogen. In this study, significantly higher mortality rates were observed for the eggs exposed to the pathogen compared to the controls. However, this method bypasses the immune adaptive response (which is in a stage of development) and the physical barriers of the eyed egg (chorion and membranes). In addition, the vertical transmission and the intra ovum presence of this bacterium in rainbow trout has not been clearly demonstrated [11,48].

*F. psychrophilum* did not grow actively in water, but its concentration remained stable up to 13 days after inoculation (Figure 3B). This was in agreement with previous studies [49,50], where the concentration of *F. psychrophilum* in stream water and sterilized natural freshwater (measured by CFU count) remained stable for 116 days [49] and for 300 days [50], respectively. However, an initial drop in the bacterial concentration was detected in [50]. In [49], the authors observed that the number of viable bacterial cells was higher (viable but non-culturable; measured by a viability assay) than the one enumerated with CFU count, suggesting that *F. psychrophilum* may undergo a starvation phase. Even after 9 months, cells were resuscitated in *Cytophaga* broth, regaining their initial morphology [49]. Similar observations have been recorded for *F. columnare* [51].

Experiments with eggs and *F. columnare* showed that the presence of nutrients (Shieh medium) had an adverse effect on egg survival, and maybe more importantly, that their optimum temperature does not match. While *F. columnare* grows well in high (above +18 °C) temperatures, this is not a suitable temperature for egg viability (the incubation of eyed eggs at temperatures higher than 12 °C can cause the development of skeletal deformities [52]). It is thus unlikely that *F. columnare* would cause problems in rainbow trout eyed eggs. However, different bacterial strains may have the ability to also grow at these lower temperatures [44], therefore the interactions between *F. columnare* and rainbow trout eggs might occur [53]. Furthermore, since this pathogen is also present in warm countries and tropical fish [54,55], and *F. columnare* has been previously found in association with Chinook salmon eggs [56], it remains relevant to study the association of bacteria and their phages in fish eggs. Indeed, *F. columnare* was isolated from all treatments (Figure S1), suggesting potential interactions in the hatchery conditions where conditions favor the presence of this bacterium.

Growth of bacteria/fungi other than the one of interest was detected (section A,B and C) and it was more prominent in experiments performed during late spring (including not published data), suggesting seasonal changes in the microbial community surrounding the chorion of the eyed eggs [57]. In addition, the lysis of bacterial cells caused by the phages releases nutrients and may stimulate the growth of other bacteria, as suggested by [58]. However, bacterial growth other than *F. psychrophilum* was detected independently of phage presence in the case of bacteria-challenged eggs (Exp. I section C, Figure 4B). Less detection of bacteria other than *F. psychrophilum* was observed in the control group for the bacterial infection. It is known that the iodine disinfection, a standard disinfection method for salmonid eyed eggs in hatcheries, does not create a sterile environment [56,59]. However, the use of higher iodine concentrations than the ones used are not recommended since this may compromise the survival of the eggs after the treatment. The growth of a background bacterial community was previously observed in phage studies in challenge experiments with *Vibrio* spp. and fish larvae [58,60]. Here, a positive effect of phages on the survival of *Vibrio*-challenged turbot and cod larvae were detected, despite a relatively high mortality caused by the background larval-associated bacterial community [36]. In our experiments, a correlation between higher mortality and the detection of other bacteria was not observed.

It is important to be aware that our experimental approach is only valid for short-term disinfection efficiency experiments, and does not consider the effects on overall survival or the hatching rate of the eyed eggs, and other factors, e.g., oxygen requirements (in salmonid eggs the hatching of the eggs happens faster in conditions of asphyxia) [61], may be influencing these parameters.

### 4.2. Rainbow Trout Eyed Eggs–Bacteriophage Interactions

The virulent phages FpV4 and FPSV-D22 targeting *F. psychrophilum* do not seem to affect the survival of rainbow trout eyed eggs (Table 2), as rainbow trout eyed eggs could tolerate the presence of bacteriophages under the tested conditions. These effects were observed for up to 71 h when phages were diluted in TYES medium (Exp. I section B), and up to 144 h when in Milli-Q water (Exp. II section B), and thus indicated that phage applications for *F. psychrophilum* control do not have a negative impact on egg survival for the tested time period. Similar results were obtained by Silva et al. [60], where the exposure of zebrafish larvae to *Vibrio* phages did not negatively affect the survival of the larvae. However, the embryo movement was not observed for a higher number of eyed eggs exposed to the phage FpV4 in crude lysate compared to the other groups (Exp. I section B), suggesting that PEG-purified solutions should be chosen over crude lysates for long-term exposures.

The qualitative and quantitative analysis of phages showed that it was possible to detect FpV4 and FPSV-D22 associated with eyed eggs after both constant (Exp. I section B) and short-term bath exposure (Exp. II section B). While the concentration of phages associated with the eggs was maintained over time during constant exposure (Figure 4A), it decreased after 24 h post-phage bath (Figure 4B) suggesting that phages do not tightly interact with the surface of the eggs. In particular, while a significant increase in the phage FpV4 concentration was detected during constant PEG-purified phage exposure, FpV4 in connection with eyed eggs was detected at very low titers 24 h after the phage bath, and could not be detected in the following samplings. The concentration of phages in the surrounding medium was constant. In contrast, FPSV-D22 in connection with the eyed eggs was detected for a longer period after the bath procedure and in a higher concentration compared to FpV4 during constant exposure experiments. These differences likely reflect differences in adherence and stability of the two phages during the interactions with the egg membranes. FpV4 belongs to the Podoviridae family with very short tails, whereas FPSV-D22 is a Siphoviridae [27,36,62] with long flexible tails, and these differences in phage morphology may affect their adherence to biotic surfaces. In addition, the time of exposure seems to represent an important variable.

Similar results were obtained with phages infecting *F. columnare* (FCL-2 and FCOV-F27): phages were detected from eggs at very low titer, while maintaining high concentrations in the surrounding medium. Further investigations are needed to shed light on this matter. Indeed, the binding of *F. columnare* phages on mucins found in mucosal surfaces have provided promising results for phage-based bacterial control and prophylaxis [63]. However, it is unclear if similar mucin glycoproteins are present on egg surfaces, which also is distinct to the mucosal secretion of fish skin. Therefore, the phages may not strictly bind to the chorion, but rather survive in the surrounding environment. Nevertheless, the presence of pathogen-targeting phages in the proximity of the eggs may prevent the bacterial infection after hatching.

### 4.3. Phages as Control Agents for F. psychrophilum in Eyed Eggs

The combined action of FpV4 and FPSV-D22 demonstrated the ability to reduce the number of bacteria associated with the eyed eggs and contained in the corresponding bath/well during the first 24 hpi (Figure 6, Phage bath no. 1: 10^9^ PFU mL^−1^). However, this controlling effect of the phages was only temporary, and the observed negative effect of the SM buffer on *F. psychrophilum* growth (more markedly for *F. psychrophilum* 950106-1/1), likely overshadowed the effects of the phage treatment after 24 h. The inhibiting effect of the buffer was thought to be related to the NaCl concentration in this buffer (0.6%). Previous studies have shown that *F. psychrophilum* can tolerate NaCl concentrations in the range 0.5–1.0%, but these properties vary among strains [29,64]. However, more studies are required to assess the potential of phage control on time scales beyond 24 h, using different incubation media that do not inhibit bacterial growth in the control cultures. The detection of *F. psychrophilum* colonies after the initial decrease in phage-treated groups may indicate the development of phage-resistant mutants (Figure 6A). In [60], zebrafish larvae (chosen as biological model system) exposed to phage VP-2 were characterized by a significantly lower mortality than the ones challenged with *Vibrio* only. The authors also observed the growth of some phage-resistant mutants of the pathogenic bacteria with a different morphology, which are generally characterized by a loss of virulence.

If *F. psychrophilum* growth was negatively affected by the SM buffer, a 10-fold increase in *F. psychrophilum* cells associated with the eyed eggs was instead detected during the 48 h of bathing in the Milli-Q water-control bath (Figure 6). Knowing that this bacterium does not grow actively in water, the reduced water flow was thought to stimulate the overgrowth of the pathogen on the egg surface. In addition, starved cells of *F. psychrophilum* have been shown to adhere to unfertilized eggs in higher numbers [65]. Moreover, no significant difference in the *F. psychrophilum* concentration was detected when including a drying step in the sampling procedure (Figure 5). All these findings suggest that these bacteria were indeed directly associated with the egg surface. Moreover, cells of *F. psychrophilum* 160401-1/5N adhered to the eyed eggs in a higher number than *F. psychrophilum* 950106-1/1 (Figure 5), suggesting that strain-specific differences in cell-adherence properties may be due to specific properties of the isolates. A previous study have shown large differences in adherence properties between different *F. psychrophilum* strains [31], but that analysis based on using polystyrene surfaces did not find different adhesion properties of the strains 160401-1/5N and 950106-1/1 used in the current study. Despite that, the ability to adhere to polystyrene surfaces is likely not directly comparable to their ability to colonize fish eggs.

### 4.4. Phages as Control Agents for F. columnare in Eyed Eggs

*F. columnare* has been found in the eggs and ovarian fluids of Chinook salmon (*Oncorhynhus tshawytscha*) [66,67]. Here, we tested if phage baths can control *F. columnare* in relation to eyed eggs, either given as prophylactic treatments or following exposure to bacteria. Although detected in the first experiments described above (Figure S1), *F. columnare* was not isolated from any of the medium or egg samples taken at any sampling time point in later experiments (Table 3 and Figure 8). This was probably caused by the experimental temperature (10 °C) in those experiments, which was too low for the bacterium. Similarly, in a study by Barnes et al. (2009) [56], *F. columnare* was found to interact with eggs, but the bacteria had no effect on salmonid egg survival at temperatures between 10–12 °C. Indeed, the adhesion, replication, and virulence characteristics of this bacterial species are strongly dependent on temperature [16,68,69], and the lack of bacterial growth in our experiment hampered the assessment of the effect of phages on the prevention of this bacterium. Yet, as an encouraging fact, neither bacterial nor phage addition had any adverse effects on egg survival. Constant phage treatments yielded 10^5^ PFU per mL titers up to 48 h. However, as mentioned above, while the optimum temperatures for *F. columnare* and salmonid eggs do not match, the results obtained in this study may be beneficial for warm water fish species, suggesting a need for similar experiments in such species.

## 5. Conclusions

To the authors’ knowledge, the present work represents the first study exploring the potential of using bacteriophages to control *Flavobacterial* pathogens in relation to salmonid eyed eggs. The results demonstrated a strong potential for short term (24 h) phage control of *F. psychrophilum* colonization of rainbow trout fry eggs. However, further studies are needed to explore if phage control can be maintained beyond 24 h and to better understand the mechanisms of interaction between flavobacteria and their phages in connection with rainbow trout eyed eggs. For example, microscopy based methods to visualize the interactions could be used.

## Figures and Tables

**Table 1:** Overview of studies focused on exploring the interactions between rainbow trout eyed eggs and *Flavobacterium* spp. bacteriophages (section B and C).

Study Name	Infection with *Flavobacterium spp.*	Type of Exposure to Phages	Phages	Type of Preparation
Exp. I Section B	No	Constant	FpV4 and FPSV-D22 (singularly)	Crude lysates and PEG-purified in sterile TYES-B
Exp. II Section B	No	4 h bath	FpV4 and FPSV-D22 (singularly)	Crude lysates
Exp. III Section B	No	30 min bath; constant	FCL-2 and FCOV-F27 (singularly)	Crude lysates
Exp. I Section C	Yes	48 h bath	FpV4 and FPSV-D22 (mixed 1:1)	PEG-purified in SM buffer
Exp. II Section C	Yes	2 h bath; constant	FCL-2	Diafiltration

**Table 2:** Exp. I and II, section B: survival of rainbow trout eyed eggs exposed to phage FpV4 and FPSV-D22. In (**A**), characteristics of the eyed eggs during constant phage exposure (Exp. I). In (**B**), characteristics of the eyed eggs after a 4 h phage bath (**B**, Exp. II). FpV4 and FPSV-D22 were diluted in sterile TYES-B. In yellow: not clear if the egg is alive (no movement and/or light turbidity); in red: the egg is dead (no movement and positive turbidity); not highlighted: the egg is alive. Time = hours of constant phage exposure in **A** and hours post-phage bath in **B**.

**(A) Constant Phage Exposure (Exp. I Section B)**													
**Time (h)**	**Evaluated** **Parameters**	**FpV4**						**FPSV-D22**			**Control**		
		**Crude Lysate**			**PEG-Purified**			**PEG-Purified**					
		**no. 1**	**no. 2**	**no. 3**	**no. 1**	**no. 2**	**no. 3**	**no. 1**	**no. 2**	**no. 3**	**no. 1**	**no. 2**	**no. 3**
**2**	Movement	+	-	+	(+)	+	+	+	+	(+)	+	+	+
	Turbidity	-	-	-	-	-	-	-	-	-	-	-	-
**27**	Movement	+	+	-	+	+	+	+	+	+	+	-	+
	Turbidity	-	-	(+)	-	-	-	-	-	-	-	-	-
**49**	Movement	+	+	-	+	+	+	+	+	+	+	+	+
	Turbidity	(+)	(+)	(+)	(+)	(+)	(+)	(+)	(+)	(+)	-	(+)	(+)
**71**	Movement	-	-	+	+	+	+	(+)	+	+	+	+	(+)
	Turbidity	+	(+)	-	(+)	(+)	(+)	-	(+)	(+)	-	-	(+)
**144**	Alive/Dead	Dead	Dead	Dead	Alive	Alive	Dead	Dead	Dead	Dead	Dead	Dead	Alive
	Hatched or not	Yes	No	No	Yes	Yes	Yes	Yes	No	Yes	No	Yes	No
**(B) Phage bath exposure (Exp. II section B)**													
**Time (h)**	**Evaluated** **Parameters**	**Crude lysate**						**Control**					
		**FpV4**			**FPSV-D22**								
		**no. 1**	**no. 2**	**no. 3**	**no. 1**	**no. 2**	**no. 3**	**no. 1**		**no. 2**		**no. 3**	
**0**	Movement	+	+	+	(+)	(+)	(+)	+		+		+	
	Turbidity	-	-	-	-	-	-	-		-		-	
**24**	Movement	(+)	(+)	(+)	+	(+)	(+)	+		+		+	
	Turbidity	-	-	-	-	-	-	-		-		-	
**46**	Movement	+	+	+	+	+	+	+		+		+	
	Turbidity	-	-	-	-	-	-	-		-		(+)	
**68**	Movement	+	(+)	+	(+)	+	+	+		+		+	
	Turbidity	-	-	-	-	-	-	(+)		(+)		(+)	
**144**	Alive/Dead	Alive	Alive	Alive	Alive	Alive	Dead	Alive		Alive		Alive	
	Hatched or not	No	Yes	Yes	No	Yes	No	Yes		No		Yes	

+ Positive to movement or turbidity; (+) Weak movement/light turbidity; - Negative to movement or turbidity.

**Table 3:** Exp. III, section B: *F. columnare* infecting phage titers in eggs and the surrounding medium (water or Shieh medium) at 0, 24, and 48 h. Phage counts for two individual samples are provided for each treatment. A “+” indicates a positive detection of phages.

(A) Exposure to Phage FCL-2								
Medium	Sample	Phage exposure	Time (h)					
			0		24		48	
			No. 1	No. 2	No. 1	No. 2	No. 1	No. 2
WATER	Well (PFU ml^−1^)	Bath	3.0 × 10^6^	9.0 × 10^5^	3.0 × 10^6^	5.0 × 10^5^	4.0 × 10^6^	3.0 × 10^6^
		Constant	9.0 × 10^7^	1.0 × 10^9^	9.0 × 10^6^	1.0 × 10^9^	3.0 × 10^7^	2.5 × 10^7^
		Control (no egg)	2.3 × 10^9^	1.0 × 10^9^	1.0 × 10^7^	1.0 × 10^7^	1.5 × 10^7^	5.0 × 10^6^
	Egg (PFU egg^−1^)	Bath	0	0	0	0	0	0
		Constant	0	2.0 × 10^1^	2.0 × 10^2^	0	9.3 × 10^1^	5.0 × 10^1^
SHIEH	Well (PFU ml^−1^)	Bath	8.0 × 10^6^	7.0 × 10^7^	2.0 × 10^8^	1.0 × 10^7^	9.0 × 10^6^	7.0 × 10^7^
		Constant	3.5 × 10^9^	2.0 × 10^9^	8.0 × 10^9^	2.0 × 10^9^	4.5 × 10^9^	5.5 × 10^9^
		Control (no egg)	3.5 × 10^9^	2.0 × 10^9^	3.5 × 10^9^	4.0 × 10^9^	2.0 × 10^9^	8.0 × 10^8^
	Egg (PFU egg^−1^)	Bath	0	0	0	0	0	0
		Constant	0	0	0	0	0	0
**(B) Exposure to Phage FCOV-F27**								
**Medium**	**Sample**	**Phage exposure**	**Time (h)**					
			**0**		**24**		**48**	
			**No. 1**	**No. 2**	**No. 1**	**No. 2**	**No. 1**	**No. 2**
WATER	Well (PFU ml^−1^)	Bath	1.5 × 10^5^	1.0 × 10^5^	+	+	+	4.0 × 10^5^
		Constant	1.3 × 10^7^	5.0 × 10^6^	3.0 × 10^6^	2.0 × 10^6^	+	2.0 × 10^6^
		Control (no egg)	1.5 × 10^7^	2.1 × 10^7^	+	3.0 × 10^6^	1.8 × 10^7^	+
	Egg (PFU egg^−1^)	Bath	0	0	0	0	0	0
		Constant	2.3 × 10^2^	0	5.0 × 10^0^	0	0	0
SHIEH	Well (PFU ml^−1^)	Bath	2.0 × 10^6^	1.0 × 10^6^	6.0 × 10^6^	7.0 × 10^6^	2.0 × 10^7^	1.0 × 10^7^
		Constant	4.5 × 10^9^	1.0 × 10^9^	2.0 × 10^9^	8.0 × 10^8^	1.5 × 10^9^	9.0 × 10^8^
		Control (no egg)	6.0 × 10^8^	5.0 × 10^8^	2.0 × 10^9^	8.0 × 10^8^	3.0 × 10^9^	2.0 × 10^9^
	Egg (PFU egg^−1^)	Bath	0	0	0	0	0	0
		Constant	0	0	0	0	0	0

## Data Availability

The data presented in this study are available in this article and in the supplementary material.

## Supplementary Materials

The following are available online at https://www.mdpi.com/article/10.3390/microorganisms9050971/s1. Figure S1: Survival of rainbow trout eggs at different temperatures with and without exposure to *F. columnare*, in either water or Shieh medium (Section A); Figure S2: Exp. II, section C: survival and hatching percentage recorded at the end of the experiment (144 h).

## Appendix A

**Table A1:** Experiments in section A concerning *F. psychrophilum.* Characteristics of sampled eggs during Exp. no. 1, no. 2, and no. 3. hpi = hours post infection. In exp. no. 1, movement and turbidity indicators of sampled eggs were not recorded at 1 and 3 hpi.

	Time (hpi)	Evaluated Parameters	+ *F. psychrophilum*			− *F. psychrophilum*		
			No. 1	No. 2	No. 3	No. 1	No. 2	No. 3
**Exp. no. 1**	21	Movement	(+)	+	+	+	+	+
		Turbidity	(+)	-	(+)	-	-	-
	25	Movement	+	+	+	+	+	+
		Turbidity	(+)	(+)	(+)	-	-	-
**Exp. no. 2**	0	Movement	+	+	+	+	+	+
		Turbidity	-	-	-	-	-	-
	24	Movement	+	-	+	+	+	+
		Turbidity	-	(+)	-	-	-	-
**Exp. no. 3**	0	Movement	+	+	+	+	+	+
		Turbidity	-	-	-	-	-	-
	24	Movement	+	-	+	+	+	+
		Turbidity	-	-	-	-	-	-

+ Positive to movement or turbidity; (+) Weak movement/light turbidity; - Negative to movement or turbidity.

**Table A2:** Experiments in section A concerning *F. psychrophilum.* Bacterial growth other than *F. psychrophilum* in sampled eyed eggs and the corresponding bath/well. A plus symbol with an orange background indicates a positive detection of bacterial colonies in TYES-B and/or on Blood-A (“-” = no growth).

	Time (hpi)	Sample (Medium Type)	+ *F. psychrophilum*			− *F. psychrophilum*		
			No. 1	No. 2	No. 3	No. 1	No. 2	No. 3
**Exp. no. 1**	**25**	Egg	n.d.	n.d.	n.d.	n.d.	n.d.	n.d.
		Well (Blood-A)	-	-	-	-	-	-
**Exp. no. 2**	**0**	Egg (TYES-A)	+	-	+	+	+	-
		Bath (Blood-A)	-			-		
	**24**	Egg (TYES-A)	-	-	-	+	+	-
		Well (TYES-A; Blood-A)	-; -	-; -	-; -	+; -	-; -	-; -
**Exp. no. 3**	**0**	Egg	n.d.	n.d.	n.d.	n.d.	n.d.	n.d.
		Bath (Blood-A)	-			-		
	**24**	Egg (TYES-A)	+	-	-	+	+	+
		Well (TYES-A)	-	-	-	+	+	+

n.d = not determined.

**Table A3:** Exp. I section B. Bacterial growth in the well of sampled eyed eggs was assessed during the experiment. A plus symbol with an orange background indicates a positive detection of bacterial colonies in TYES-B and/or on Blood-A (“-” = no growth). *F. psychrophilum* was not detected.

Time (h)	Medium Type	+ FpV4						+ FPSV-D22			Control		
		Crude Lysate			PEG-Purified			PEG-Purified					
		No. 1	No. 2	No. 3	No. 1	No. 2	No. 3	No. 1	No. 2	No. 3	No. 1	No. 2	No. 3
2	TYES-A	-	-	-	-	-	-	-	-	-	-	-	-
	Blood-A	-	-	-	-	-	-	-	-	-	-	-	-
27	TYES-A	+	-	-	+	-	-	-	-	-	-	-	-
	Blood-A	+	-	-	+	-	-	-	-	-	-	-	-
49	TYES-A	-	-	-	-	-	-	-	-	-	-	-	-
	Blood-A	+	-	+	-	-	-	+	-	+	-	+	-
71	TYES-A	n.d.			n.d.			n.d.			n.d.		
	Blood-A	-	-	-	-	-	-	-	-	-	-	-	-
144	TYES-A	n.d.			n.d.			n.d.			n.d.		
	Blood-A	-	-	-	-	-	+	+	-	-	+	-	-

n.d. = not determined.

**Table A4:** Exp. II section B. Bacterial growth in the well of sampled eyed eggs was assessed during the experiment. A plus symbol with an orange background indicates a positive detection of bacterial colonies in TYES-B and/or on Blood-A (“-” = no growth). *F. psychrophilum* was not detected.

Time (h)	Medium Type	+ Crude Lysate						Control		
		+ FpV4			+ FPSV-D22					
		No. 1	No. 2	No. 3	No. 1	No. 2	No. 3	No. 1	No. 2	No. 3
0 *	TYES-A	-			-			-		
	Blood-A	-			-			-		
24	TYES-A	-	-	-	-	-	-	-	-	-
	Blood-A	-	-	-	-	-	-	-	-	-
46	TYES-A	-	-	-	-	-	-	-	-	-
	Blood-A	-	-	-	-	-	-	-	-	-
68	TYES-A	n.d.			n.d.			n.d.		
	Blood-A	-	-	-	-	-	-	-	+	-
144	TYES-A	n.d.			n.d.			n.d.		
	Blood-A	-	-	+	+	-	-	+	+	-

* Bath content; n.d. = not determined, not sampled.
