# Supplementary material for: Interactions between Rainbow Trout Eyed Eggs and Flavobacterium spp. Using a Bath Challenge Model: Preliminary Evaluation of Bacteriophages as Pathogen Control Agents
Source: Microorganisms. 2021 Apr 30;9(5):971. doi: 10.3390/microorganisms9050971 (PMC8146780; doi:10.3390/microorganisms9050971)
Supplement: Supplementary file 1 [file microorganisms-09-00971-s001.zip › microorganisms-1145052-supplementary.pdf]

Supplementary Material: Interactions between Rainbow Trout Eyed Eggs and *Flavobacterium* spp. Using a Bath Challenge Model: Preliminary Evaluation of Bacteriophages as Pathogen Control Agents

Valentina L. Donati [1,*], Inger Dalsgaard [1], Anniina Runtuvuori-Salmela [2], Heidi Kunttu [2], Johanna Jørgensen [3], Daniel Castillo [3,4], Lotta-Riina Sundberg [2], Mathias Middelboe [3] and Lone Madsen [1]

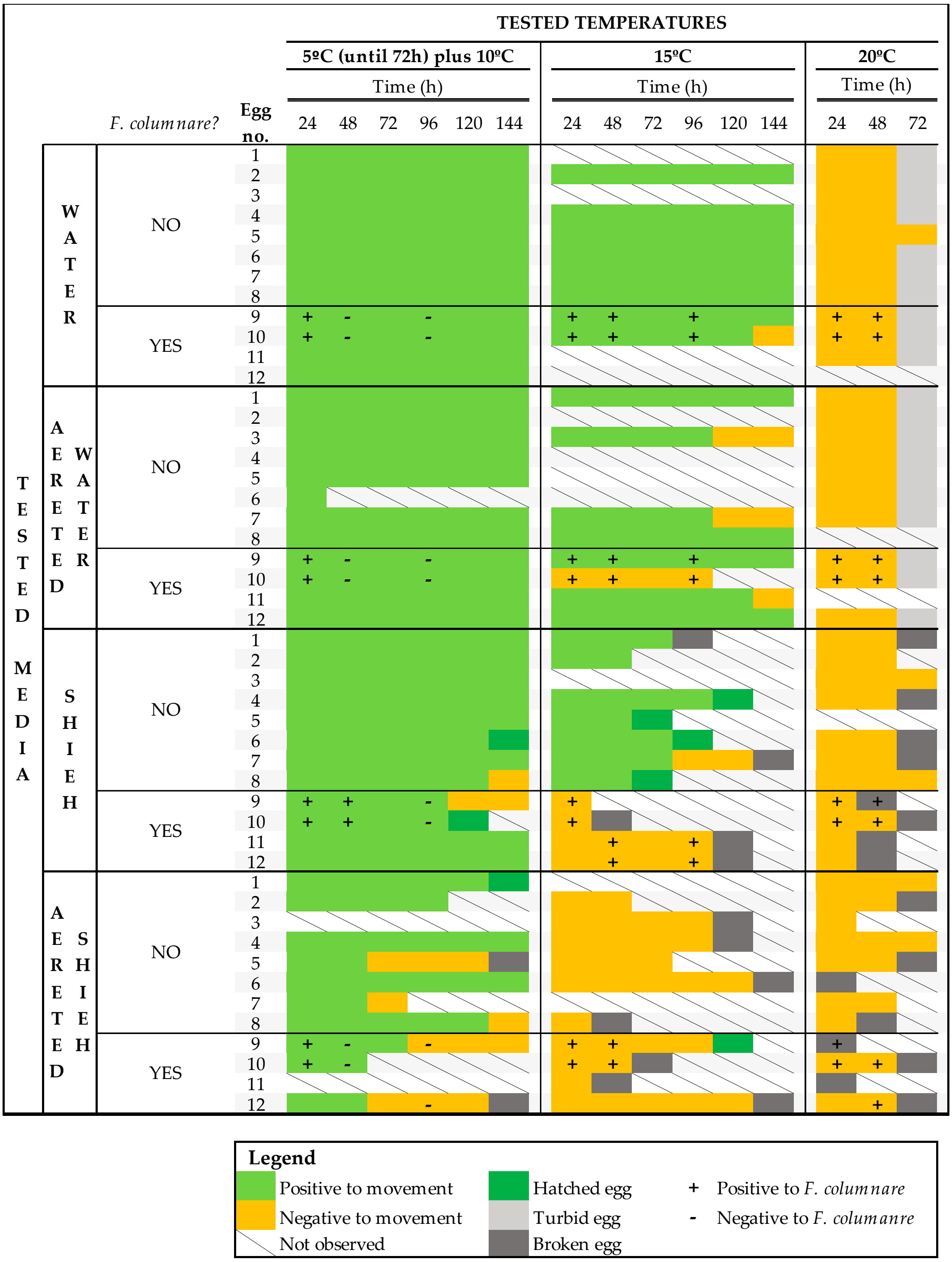

Figure S1. Survival of rainbow trout eggs at different temperatures with and without exposure to *F. columnare*, in either water or Shieh medium (Section A).

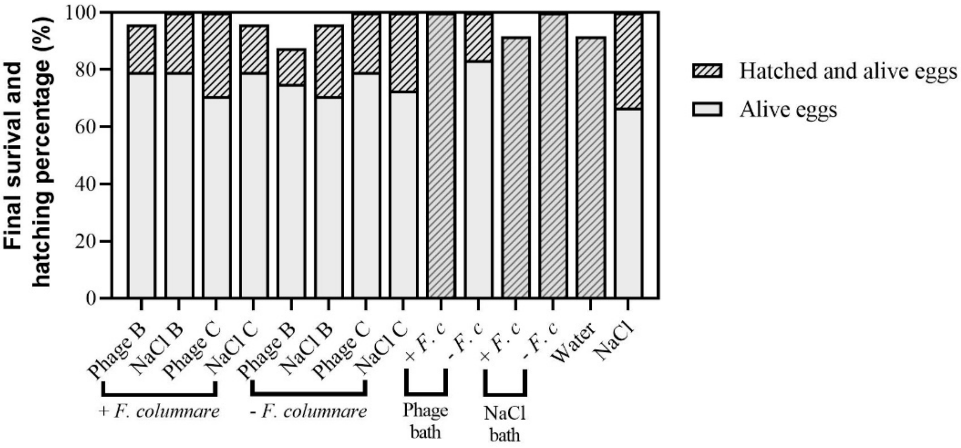

Figure S2. Exp. II, section C: survival and hatching percentage recorded at the end of the experiment (144 h). B: bath exposure, C: constant exposure, *F. c* = *F. columnare* B185.
